# New 3-Cyano-2-Substituted Pyridines Induce Apoptosis in MCF 7 Breast Cancer Cells

**DOI:** 10.3390/molecules21020230

**Published:** 2016-02-18

**Authors:** Ahmed Malki, Mona Mohsen, Hassan Aziz, Ola Rizk, Omaima Shaaban, Mohamed El-Sayed, Zaki A. Sherif, Hayam Ashour

**Affiliations:** 1 Biomedical Science Program, Department of Health Sciences, College of Art and Sciences, Qatar University, Doha 2713, Qatar; mmohsen@qf.org.qa (M.M.); hassan.aziz@qu.edu.qa (H.A.); 2 Department of Pharmaceutical Chemistry, Faculty of Pharmacy, Alexandria University, Alexandria 21521, Egypt; olarizk@yahoo.com (O.R.); omimagaber@yahoo.com (O.S.); 3 Department of Analytical and Pharmaceutical Chemistry, Faculty of Pharmacy & Drug Manufacturing, Pharos University, Alexandria, 21311, Egypt; 4 Biochemistry Department, Faculty of Science, Alexandria University, Alexandria 21521, Egypt; mmaelsayed@yahoo.com; 5 Department of Biochemistry and Molecular Biology, Howard University, College of Medicine, Washington, DC 20059, USA; zakisherif@Howard.edu

**Keywords:** 3-cyanopyridines, alkoxy substituents, MCF-7, apoptosis, p53, VEGF

## Abstract

The synthesis of new 3-cyano-2-substituted pyridines bearing various pharmacophores and functionalities at position 2 is described. The synthesized compounds were evaluated for their *in vitro* anti-cancer activities on five cancer cell lines using 5-FU as reference compound. The results revealed that the benzohydrazide derivative **9a** induced growth inhibition in human breast cancer cell line MCF-7 with an IC_50_ value of 2 μM and it showed lower cytotoxicity on MCF-12a normal breast epithelial cells. Additionally, **9a** induced apoptotic morphological changes and induced apoptosis in MCF-7 in a dose and time-dependent manner according to an enzyme linked immunosorbent apoptosis assay which is further confirmed by a TUNEL assay. Flow cytometric analysis indicated that **9a** arrested MCF-7 cells in the G1 phase, which was further confirmed by increased expression of p21 and p27 and reduced expression of CDK2 and CDK4. Western blot data revealed significant upregulation of the expression of p53, Bax, caspase-3 and down-regulation of Bcl-2, Mdm-2 and Akt. Additionally, **9a** increased the release of cytochrome c from mitochondria to cytoplasm which provokes the mitochondrial apoptotic pathway while it showed no significant change on the expression of the death receptor proteins procaspase-8, caspase-8 and FAS. Furthermore, **9a** reduced the expression of phospho AKT and β-catenin in dose dependent manner while inhibiting the expression of migration-related genes such as matrix metalloproteinase (MMP)-9 and vascular endothelial growth factor (VEGF). Our findings suggest that compound **9a** could be considered as a lead structure for further development of more potent apoptosis inducing agents with anti-metastatic activities.

## 1. Introduction

Breast cancer is a complex disease that comprises heterogenous tumors associated with distinctive histological patterns and different clinical characteristics [1]. An estimated 1.7 million women will be diagnosed with breast cancer in 2020, a 26% increase from current levels [2]. Approximately 30% of the women diagnosed with early-stage disease subsequently progress to metastatic breast cancer (MBC), for which few therapeutic regimens exist [3]. Many factors contribute to the metastasis of tumor cells; these include molecular signaling pathways, and expression of metastasis-related genes such as matrix metalloproteinases (MMPs) [4]. Despite the advances in therapeutic modalities, MBC remains incurable, with an estimated survival rate of about 23% over 5 years [5]. Chemotherapy resistance is also a consistent obstacle in the management of breast cancer, where many of the initially responsive tumors relapse and develop resistance to diverse chemotherapeutic agents [6]. Consequently, new agents with low susceptibility to common drug resistance mechanisms are urgently needed for the management and treatment of various forms of breast cancer.

The anticancer activity of most cytotoxic therapies which are currently used in the clinical management of cancer patients including radiotherapy is based on their ability to induce cell death programs such as apoptosis [7]. Apoptosis is an evolutionary conserved process characterized by a series of morphological and biochemical changes, including cell shrinkage, nuclear DNA fragmentation and membrane blebbing [8].

**Figure 1:**
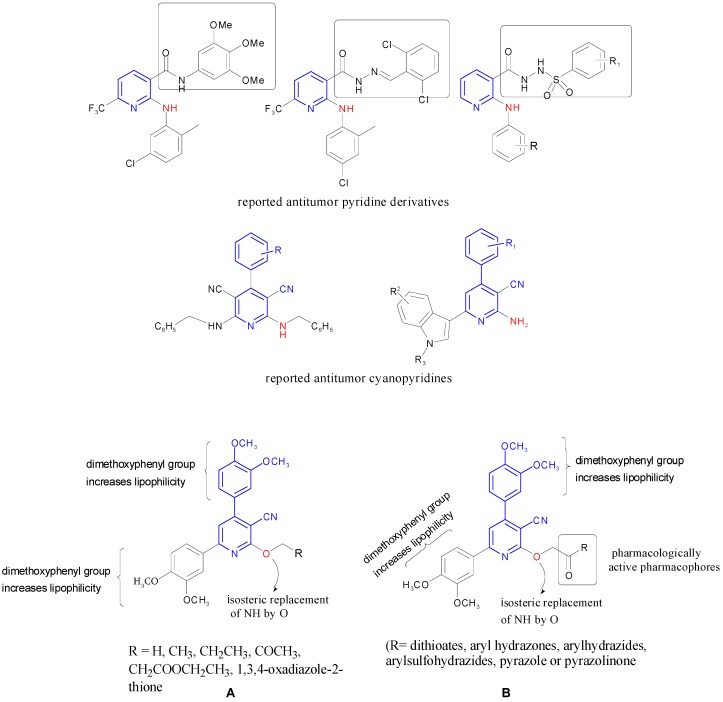
Chemical structure of reported pyridines and cyanopyridines endowed with anticancer and apoptosis-inducing activities and the synthesized compounds (**A**,**B**).

Pyridine derivatives have always constituted a subject of great interest due to the ubiquity of pyridine in Nature and the extensive presence in the skeletal backbone of many therapeutic agents. These derivatives were reported to possess cytotoxic [9], and anticancer activities [10,11,12,13,14,15,16,17,18] in addition to topoisomerase I, II [19,20,21] and Src inhibitory activities [22,23,24]. Penclomedine (PEN1, 3,5-dichloro-4,6-dimethoxy-2-trichloromethylpyridine), is an antitumor agent which was selected for clinical development by the NCI based on its selective antitumor activity against certain carcinomas [25]. Moreover, triapine (3-aminopyridine-2-carboxaldehyde thiosemicarbazone) is a potent ribonucleotide reductase inhibitor that has shown potent antiproliferative activity against several cancer cell lines [26]. Furthermore, particular interest has been focused on pyridine carbonitriles due to their remarkable *in vitro* anticancer activity against a wide range of cell lines (Figure 1) [27,28,29,30]. Consequently, pyridine carbonitrile remains a promising template for the design of a new category of chemotherapeutic agents.

Inspired by the abovementioned findings and in continuation of our efforts linked to discovering and exploring novel lead heterocyclic structures as potent chemotherapeutic agents [31,32,33,34], new derivatives of 3-cyano-2-substituted pyridines were synthesized for evaluation of their *in vitro* anticancer activity. A literature survey revealed that incorporation of alkoxy substituents (methoxy and/or aryloxy moieties) results in significant enhancement of antitumor activity due to magnification of compounds’ lipophilicity [35,36]. Accordingly, the target compounds were designed so as to comprise 3,4-dimethoxyphenyl groups at positions 4 and 6. Moreover to the best of our knowledge, 2-substituted alkoxycyanopyridines are seldom reported in the literature. Therefore, it was planned to include variable substituents at position 2, linked to the cyanopyridine scaffold through a methyleneoxy or acetyloxy spacer (**A** and **B**, Figure 1). Such substituents were selected so as to offer variable electronic, lipophilic and steric environment that could influence the targeted biological activity. The substituents include either alkyl groups of different length or biologically active pharmacophores that are believed to be responsible for the biological significance of some reported anticancer agents such as benzohydrazides [37,38] benzosulfohydrazides [10], dithioates [39,40] and arylhydrazones [41,42,43]. In addition, incorporation of heterocyclic groups such as pyrazoles and 1,3,4-oxadiazoles (**B**, Figure 1) was considered as an interesting structure variation that might impose an impact on the potential biological activities owing to their documented chemotherapeutic activity [44,45,46,47,48].The *in vitro* antiproliferative activity of the newly synthesized compounds was investigated against five cancer cell lines and the effect of the most promising compound on apoptosis and expression of proteins related to cell cycle pathways was also evaluated.

## 2. Results and Discussion

### 2.1. Chemistry

**Scheme 1:**
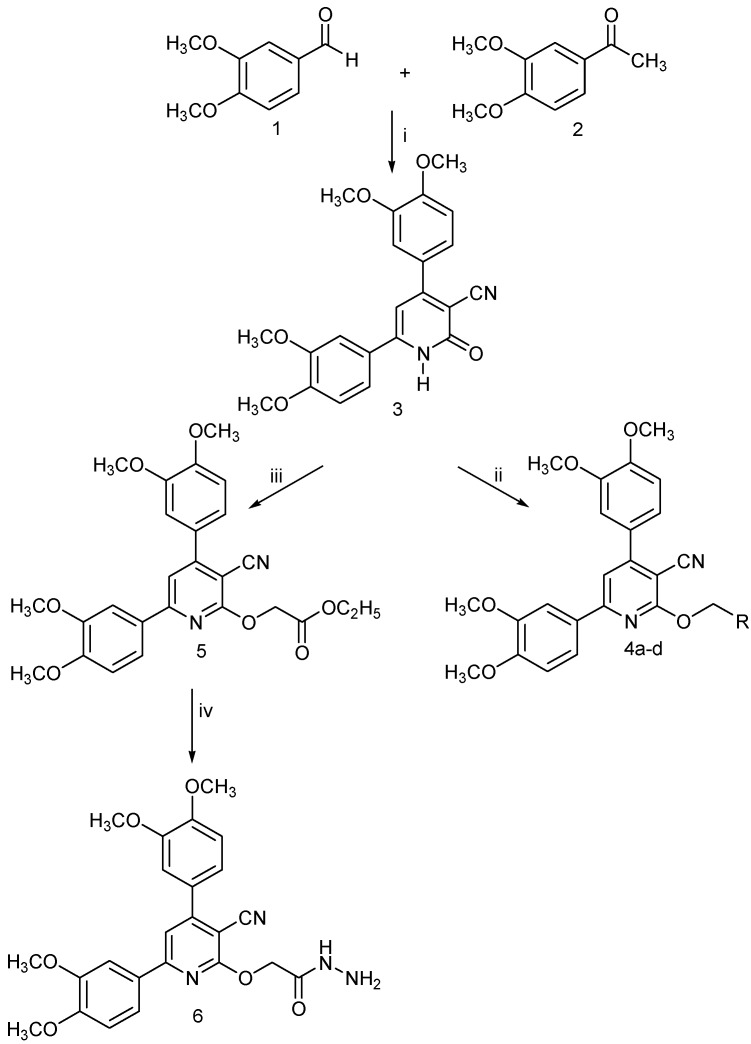
Synthesis of the target compounds **4**, **5** and the key intermediate **6**. For **4a**–**d**: a, R = H; b, R = CH_3_; c, R = CH_2_CH_3_; d, R = COCH_3_; *Reagents and conditions*: (i) CNCH_2_COOEt/anhydrous CH_3_COONH_4_/EtOH/reflux; (ii) the appropriate alkyl halide/anhydrous K_2_CO_3_/dry acetone/reflux; (iii) BrCH_2_COOEt/anhydrous K_2_CO_3_/dry acetone/reflux; (iv) N_2_H_4_ 98%/EtOH/reflux.

**Scheme 2:**
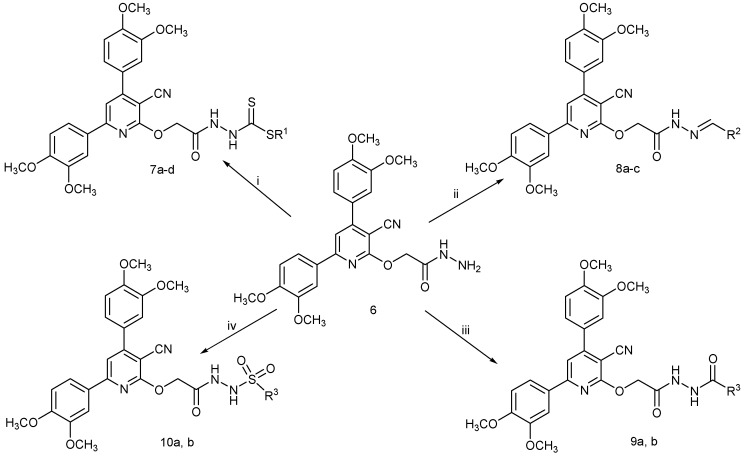
Synthesis of the target compounds **7**–**10**. For **7**: a, R^1^ = CH_3_; b, R^1^ = C_2_H_5_; c, R^1^ = 4-ethylmorpholine; d, R^1^ = 4-ethylpiperidine; **8** : a, R^2^ = 4-OCH_3_C_6_H_4_; b, R^2^ = 3,4-OCH_3_C_6_H_3_; c, R^2^ = 3,4,5-OCH_3_C_6_H_2_; **9**,**10**: a, R^3^= C_6_H_5_; b, R^3^= 4-OCH_3_C_6_H_4_; *Reagents and conditions*: (i) CS_2_/KOH/DMF/the appropriate alkyl halide/RT; (ii) the appropriate aldehyde/EtOH/CH_3_COOH/reflux; (iii) benzoyl or *p*-methoxy-benzoyl chloride/dry pyridine/RT; (iv) benzenesulfonyl or *p*-methoxybenzenesulfonyl chloride/dry pyridine/RT.

**Scheme 3:**
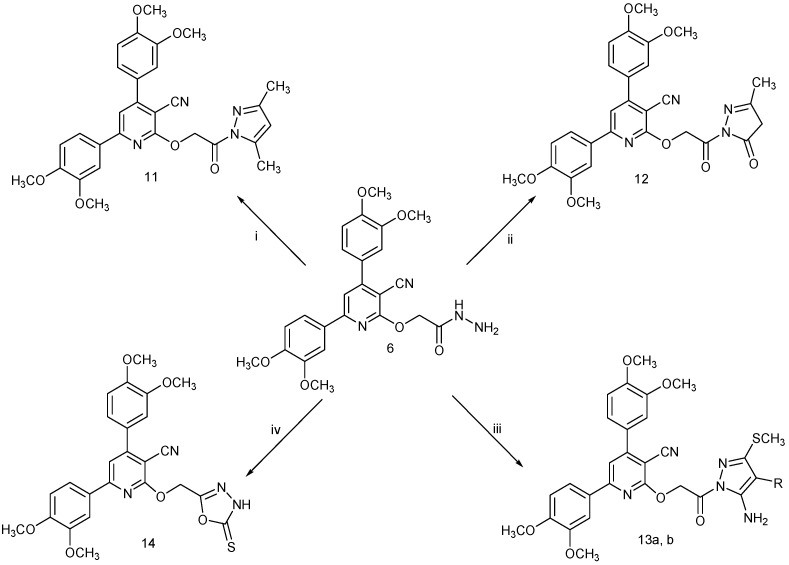
Synthesis of the target compounds **11**–**14**. For **13**: a, R = COOCH_3_; b, R = CN; *Reagents and conditions*: (i) CH_2_(COCH_3_)_2_/EtOH/gl. CH_3_COOH/reflux; (ii) CH_3_COCH_2_COOC_2_H_5_/EtOH/gl. CH_3_COOH/reflux; (iii) methyl 2-cyano 3,3-bis(methylsulfanyl)acrylate or 2-(bis(methylsulfanyl)-methylene)malononitrile/DMF/reflux; (iv) CS_2_/KOH/EtOH/reflux.

The synthetic strategies adopted for the synthesis of the intermediate and target compounds are depicted in Scheme 1, Scheme 2 and Scheme 3. In Scheme 1, the cyanopyridinone **3** was prepared according to the Al-Saadi procedure [49] via a one-pot multicomponent reaction of 3,4-dimethoxybenzaldehyde (**1**), 3,4-dimethoxyacetophenone (**2**), an excess of ammonium acetate and ethyl cyanoacetate in boiling ethanol. Heating the cyanopyridinone **3** with different alkyl halides in absolute ethanol using sodium ethoxide as a basic catalyst according to the Kornblum procedure [50] failed to afford the target O-alkylated derivatives **4a**–**d**. However, such compounds were successfully prepared by heating the cyanopyridinone **3** with the appropriate alkyl halide in acetone in the presence of anhydrous K_2_CO_3_. Similarly, refluxing **3** with ethyl bromoacetate in dry acetone containing anhydrous K_2_CO_3_ yielded the corresponding ethyl acetate ester **5**. Reaction of the ester **5** with hydrazine hydrate in refluxing ethanol resulted in the formation of the corresponding acetohydrazide **6** which was employed as key intermediate for synthesis of the target compounds presented in Scheme 2.

**Figure 2:**
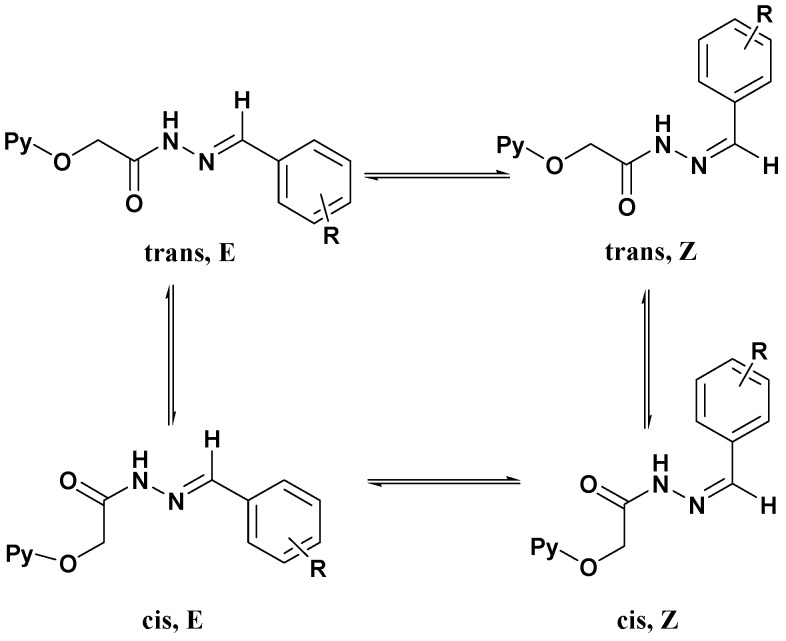
*E/Z* geometrical isomers and *cis/trans* conformers of compounds **8a**–**c**.

In Scheme 2, the hydrazide **6** was conveniently converted to the dithioate esters **7a**–**d** by reaction with carbon disulfide in DMF in presence of KOH, followed by addition of the appropriate alkyl halide at room temperature. Condensation of the hydrazide **6** with the appropriate aldehyde in boiling ethanol containing few drops glacial acetic acid afforded the respective azomethines **8a**–**c**. It should be noted down here that compounds having the arylidene–hydrazide structure may exist as *E*/*Z* geometrical isomers about the C=N bond and as *cis/trans* amide conformers at the CO–NH moiety (Figure 2) [51,52]. In this respect, the ^1^H-NMR spectra of these compounds in DMSO-*d*_6_ confirmed their existence as *E* geometrical isomers, which coincides with the literature findings for analogous compounds containing the imine (C=N) functionality [52]. On the other hand, further interpretation of their ^1^H-NMR DMSO-*d*_6_ spectra revealed the presence of three sets of signals at δ 5.09–5.11, 5.60–5.63, 7.96–8.0, 8.18–8.22 and δ 11.58–11.76, 11.62–11.80, attributed to the OCH_2_, N=CH and CO–NH groups of the *cis* and *trans* conformers, respectively. According to the literature, the upfield signals of the N=CH and CONH protons were assigned to the *cis* conformer of the amide structure, whereas, the downfield peaks were due to the *trans* conformer [53]. Treatment of the hydrazide **6** with benzoyl chloride or *p*-methoxybenzoyl chloride in pyridine at room temperature furnished the corresponding benzohydrazides **9a**,**b**. Similarly, reaction of **6** with benzenesulfonyl chloride or *p*-methoxy-benzenesulfonyl chloride gave rise to the corresponding benzenesulfohydrazides **10a**,**b** in good yield.

In Scheme 3, condensation of the hydrazide **6** with acetylacetone or ethyl acetoacetate in ethanol/glacial acetic acid mixture (2:1) furnished the corresponding 3,5-dimethylpyrazole **11** and the pyrazolinone derivative **12**, respectively. Heating **6** with methyl 2-cyano-3,3-bis(methyl-sulfanyl)acrylate or [bis(methylsulfanyl)methylene]malononitrile in dry DMF following reported reaction conditions [54] yielded the proposed 5-amino-3-methylsulfanyl-4-substituted pyrazolyl derivatives **13a**,**b** in moderate yield. On the other hand, refluxing **6** with carbon disulphide in ethanolic potassium hydroxide afforded the oxadiazolyl derivative **14**.

### 2.2. Biological Evaluation

#### 2.2.1. Effect of the Synthesized Compounds on Cell Viability of Different Cancer Cell Lines

**Figure 3:**
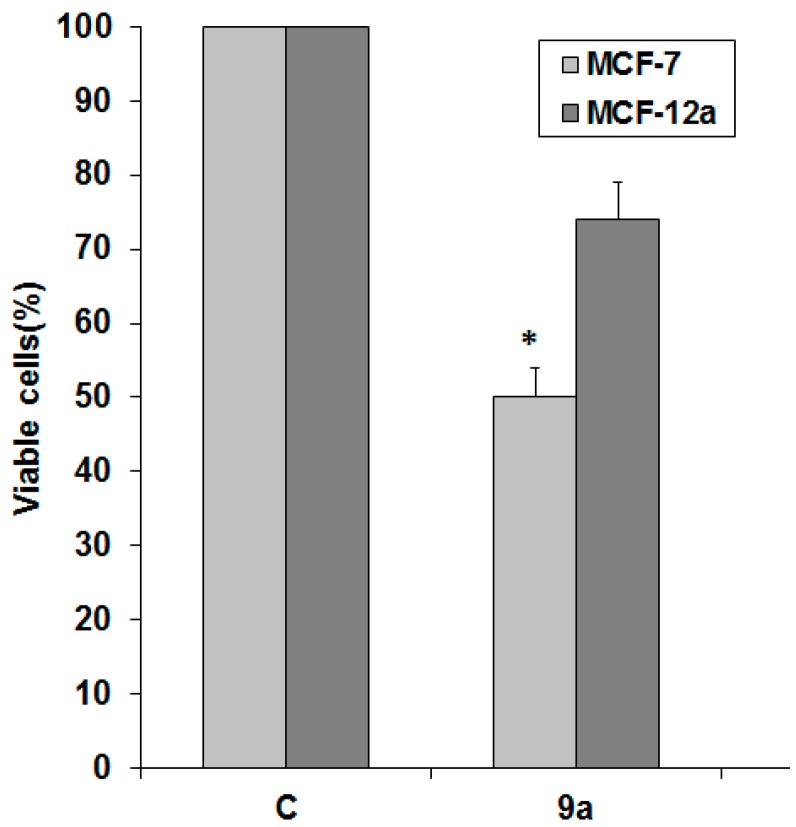
Cytotoxicity of compound **9a** on human breast cancer cells MCF-7 and non-tumorigenic breast epithelial cells MCF-12a. WST-1 Assay was performed on MCF-7 and MCF-12a after treatment with 2 μM of compound **9a** for 24 h. Each data point was an average of results from three independent experiments performed in triplicate and presented as M ± SD. Significant differences between the control and test compounds are indicated by * (* *p* ˂ 0.05).

The synthesized compounds were screened for their cytotoxicity on five cancer cell lines, namely SK-OV-3, MCF-7, MDA-MB-231, HCT-116 and RKO, using a WST colorimetric assay. The assay principle is based upon the reduction of the tetrazolium salt WST-1 to formazan by cellular mitochondrial dehydrogenases present in viable cells. The results revealed that most of the tested compounds showed higher cytotoxicity than 5-FU on SK-OV-3 cells and among them compound **7c** was the most potent as it displayed an IC_50_ value of 5.12 μM compared to 5-FU which showed an IC_50_ value of 32.19 μM. Meanwhile, compound **9a** (IC_50_ = 2.04 μM) was more potent than 5-FU (IC_50_ = 7.06 μM) against MCF-7 cells, whereas compounds **7c** and **10b** (IC_50_ = 7.1 and 8.12 μM, respectively) were nearly equipotent to 5-FU. Concerning the activity against MDA-MB-231, none of the test compounds showed remarkable effect on the viability of MDA-MB 231 cells. Moreover, the obtained data showed that compounds **4c**, **4d**, **7b**, **7d** and **9a** displayed a significant effect (IC_50_ = 7.12–13.14 μM) on the viability of HCT-116 cells compared to 5-FU and among them compound **9a** displayed the highest activity as it showed an IC_50_ value of 7.12 μM compared to 5-FU which showed an IC_50_ value of 12.19 μM. Additionally, **9a** and **9b** (IC_50_ = 8.22 and 9.95 μM, respectively) were more potent than 5-FU (IC_50_ = 20.18 μM) on RKO cells (Table 1). Taking the IC_50_ values as reliable criteria for comparison of cytotoxicity of the test compounds, it could be clearly recognized that cyanopyridines attached to heterocyclic moieties at the 2-position through acetyloxy or methyleneoxy spacers (compounds **11**–**14**) were nearly inactive compared to cyanopyridines attached to alkyl, dithioate, arylhydrazone, benzohydrazide or benzosulfohydrazide groups (compounds **4**–**10**) which showed sigificant cytotoxicity on the tested cell lines. Among the latter compounds the benzohydrazide derivative **9a** emerged with the lowest IC_50_ in this study against MCF-7 cells compared to 5-FU. The level of cytotoxicity of compound **9a** on MCF-12a normal breast epithelial cells was also investigated and the IC_50_ was found to be 7.5 ± 0.25 μM. Moreover, MCF-12a cells were treated with 2 μM **9a** for 24 h and the results (Figure 3) indicated that 2 μM of **9a** reduced cell viability to approximately 50% in MCF-7 cells compared to approximately 74% in MCF-12a cells. The significance of viability between MCF-7 and MCF-12a cells was calculated using SPSS non-parametric analysis of two independent tests and the results showed statistical significance of p value 0.001. Based on the fact that **9a** had less cytotoxic effects on normal breast epithelial cells it was subjected to further studies.

#### 2.2.2. Compound **9a** induces apoptosis in MCF-7 cells

##### Morphological Changes

**Figure 4:**
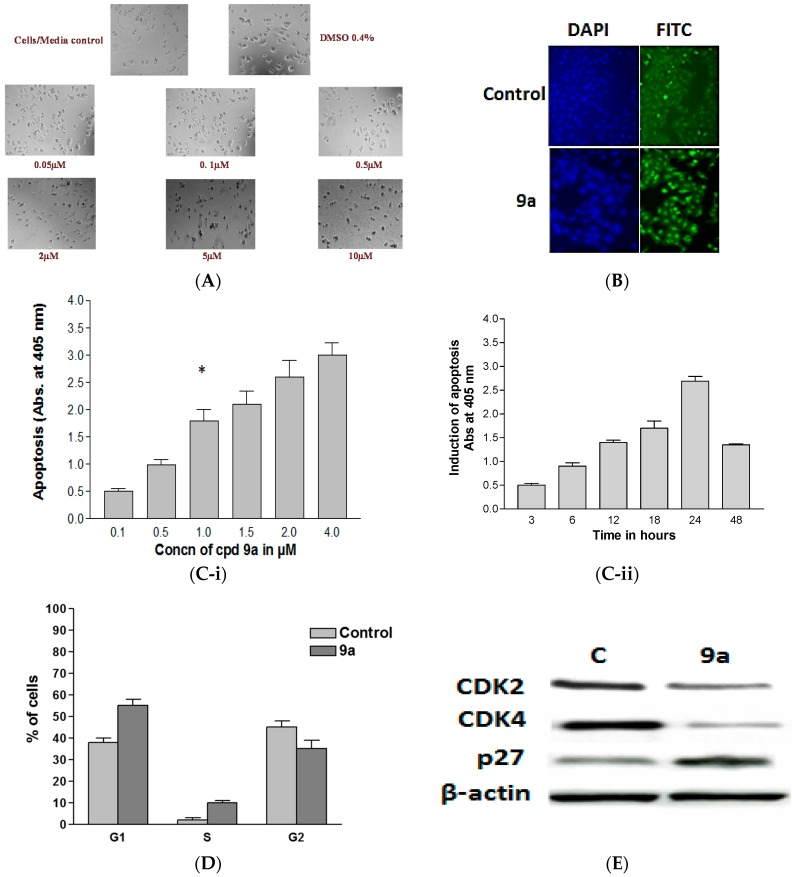
Compound **9a**-induced apoptosis in MCF-7 cells. (**A**) Morphological changes in MCF-7 cells were examined after treatment with 0.05, 0.1, 0.5, 2, 5 and 10 μM of compound **9a** for 24 h. Compound **9a** induces apoptotic death including marked apoptotic bodies and cell shrinkage in a concentration dependent manner. Untreated cells were used as a negative control. Treatment of MCF-7 cells with 0.4% DMSO did not show any cell death; (**B**) A TUNEL assay was used to confirm induction of apoptosis in MCF-7 cells. Cells were treated with 2 μM of **9a** for 24 h. Untreated cells act as control. Lack of staining in control cells shows that the cells are actively proliferating, *i.e*., no apoptotic cell death and induction of apoptosis in treated cells was confirmed by the appearance of TUNEL-positive cells; (**C**) ELISA assay apoptotic cell detection. Effect of **9a** on apoptosis induction in MCF-7 cells after treatment with different concentrations (i) and different time intervals (ii). Data are presented as mean ± SD (*n* = 3). Significant differences between the control and test compounds are indicated by * (* *p* ˂ 0.05); (**D**) Percent of distribution of cell cycle phases of MCF-7 breast cancer cells after treatment with 2 μM of **9a** for 24 h. Each experiment was performed in triplicate. Data are presented as mean ± SD (*n* = 3); (**E**) Western blot analysis was carried out to quantify the expression levels of p27, CDK2 and CDK4 in treated and non-treated control cells. β-actin was used as a loading control.

The morphological changes in MCF-7 cells were followed up after treatment with different concentrations of compound **9a** (0.05, 0.1, 0.5, 2, 5 and 10 μM) for 24 h. At 0.05, 0.1 and 0.5 μM, no morphological changes were observed, however upon treating the cells with higher concentrations of 2, 5 and 10 μM, apoptotic features were observed, including marked apoptotic bodies and cell shrinkage in a concentration dependent manner (Figure 4A). Untreated cells were used as a negative control. Treatment of MCF-7 cells with DMSO 0.4% did not show any characteristics of apoptosis in the MCF-7 cells.

##### TUNEL Assay

To ascertain induction of apoptosis of compound **9a** on MCF-7 cells, the results were confirmed by immune histochemical analysis using a TUNEL assay. TUNEL assay involves labeling of the 3'-hydroxyl DNA ends generated during DNA fragmentation by means of rTdT and labeled dUTP. The localized fluorescence of apoptotic cells (labeled-dUTP) in a blue background (DAPI) for nuclei staining or green background (FITC) was visualized using fluorescence microscope. The assay results (Figure 4B) revealed the presence of nuclear condensation and TUNEL-positive cells (stained cells) compared to control group.

##### Enzyme Linked Immunosorbent Apoptosis Assay

Compound **9a** was further investigated for induction of apoptosis in MCF-7 cells using Cell Death Detection ELISA^PLUS^ which is used for the quantitative *in vitro* determination of cytoplasmic histone-associated DNA fragments (mono- and oligonucleosomes) in apoptosing cells.

The results revealed that induction of apoptosis increased in a dose dependent fashion upon treating MCF-7 cells with different concentrations of **9a** ranging from 0.1 to 4 μM (Figure 4C-i), additionally, apoptotic induction in MCF-7 cells increases with increasing the exposure time starting from 3 h to 48 h (Figure 4C-ii) with optimum value at 24 h.

##### Flow Cytometric Analysis

Flow cytometry is considered one of the specific tools for the investigation of molecular and morphological events occurring during cell death and cell proliferation [55]. Therefore, the effect of compound **9a** on cell cycle progression was studied by flow cytometry in propidium iodide (PI) stained cells. The percent of cells in G1, S, G2 phases was determined after treating cells with 2 μM of **9a** for 24 h (Figure 4D). The results revealed a significant increase in the percent of G1 population (54%) compared to control group (37%). Thus, it can be concluded that compound **9a** inhibits the proliferation of MCF-7 cells and the growth inhibition is associated with induction of G1 phase arrest. p27 is considered as an important regulator of cell cycle [56]. To determine whether compound **9a**-induced G1 arrest was associated with increased p27 level, and to study the effect on the expression of CDK2 and CDK4 involved in cell cycle progression, cells were analyzed for the expression of these proteins using western blot analysis after treatment with 2 μM of **9a** for 24 h. The results (Figure 4E) revealed that compound **9a** increased the expression of p27 and decreased the expression of both CDK2 and CDK4.

##### Impact of **9a** on Apoptotic Signaling Pathways

**Figure 5:**
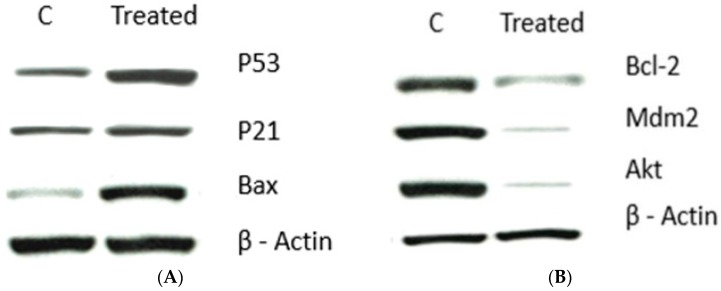

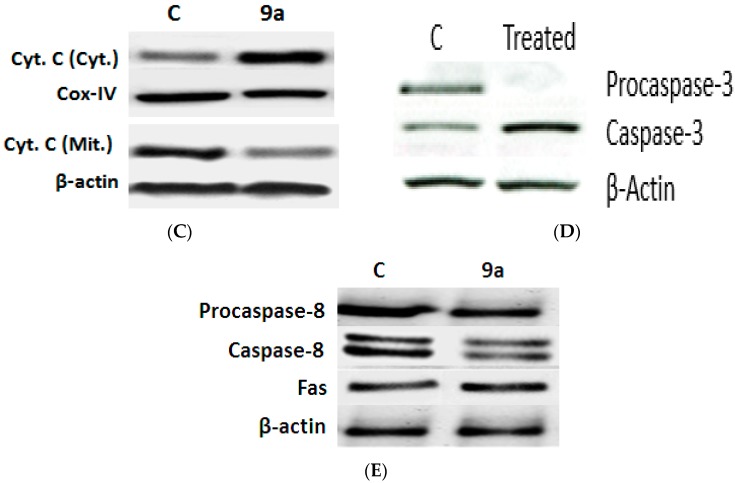
Compound **9a**-modulated apoptotic and survival signaling in MCF-7 cells. (**A**) Compound **9a** up-regulated p53, p21 and Bax. Cells were treated with 2 μM of **9a** for 24 h. The MCF-7 cell lysates were collected and the expression of p53, p21 and Bax was studied by western blot analysis using specific antibodies in treated and non-treated control cells. β-actin was used as a loading control; (**B**) Compound **9a** down-regulates Bcl-2, MDM-2 and Akt levels in MCF-7 cells. Cells were treated with 2 μM **9a** for 24 h. The cell lysates were collected and the expression of Bcl-2, MDM-2 and AKT was studied by western blot analysis using specific antibodies in treated and non-treated control cells. β-actin was used as a loading control; (**C**) Total proteins in cytosol and mitochondrial fraction were extracted and western blotting analysis was applied to determine the protein levels of cytochrome c in treated and non-treated cells; (**D**) Compound **9a** up-regulates the level of caspase-3 in MCF-7 Cells. Cells were treated with 2 μM **9a** for 24 h, cells were lysed and total protein was collected and subjected to western blot analysis to determine pro-caspase and caspase-3 proteins expression levels in treated and non-treated control cells. β-actin was used as a loading control; (**E**) The cell lysates were collected and the expression levels of FAS, procaspase-8 and caspase-8 was studied by western blotting analysis using specific antibodies, β-actin was used as loading control.

To further investigate the underlying pathways of **9a**-induced apoptosis, we evaluated the expression of some apoptotic and survival proteins involved in apoptosis such as p53, p21, Bax and antiapoptotic proteins such as Bcl-2 in addition to protein kinases e.g., AKT. The tumor suppressor protein p53 plays a critical role in inducing cell cycle arrest or apoptosis through its transcriptional activity [57]. Activation of p53 induces expression of cell cycle regulators such as p21WAF1 which is considered as an important regulator of cell cycle [58]. Moreover, activation of p53 leads to activation of the intrinsic pathway by increasing the expression of many pro-apoptotic proteins such as Bax and Bid. After reception of the death signal, Bax; a Bcl-2 family protein, induces cell death through disruption of mitochondrial permeability and subsequent release of cytochrome c where it engages in a cascade of interactions that leads to the execution stage of apoptosis [59]. In this study, the obtained results revealed that compound **9a** increased the level of p53, p21 and Bax in MCF-7 cells treated with the IC_50_ (2 μM) of compound **9a** for 24 h compared with untreated cells used as a control (Figure 5A). It is known that interactions between the pro- and antiapoptotic members of Bcl-2 family integrate diverse upstream signals to determine the cellular response. Apoptosis occurs through competitive dimerization between the two protein groups [60]. Accordingly, an increased ratio of Bax to Bcl-2 leads to programmed cell death. Therefore, we analyzed the level of Bcl-2 protein following treatment of MCF-7 cells with 2 μM **9a** for 24 h and the obtained data portrayed that the level of Bcl-2 protein was decreased (Figure 5B). Mouse Double Minute 2 Homolog (MDM2) and Akt are negative regulators of p53 and the apoptotic process. Therefore, we evaluated the effect of **9a** on these regulators. The obtained data showed that **9a** decreased levels of both MDM2 and Akt (Figure 5B). Cytochrome c, a component of the mitochondrial electron transfer chain, is released into the cytosol during the early phases of apoptosis. Therefore the accumulation of mitochondrial cytochrome c release in the cytosol was determined by immunoblotting of the cell lysate of **9a** treated cells. Data from western blot analysis (Figure 5C) showed that treatment of MCF-7 cells with 2 μM **9a** for 24 h increased the expression of cytochrome c in cytoplasm and reduced its expression in mitochondria (Figure 5C). In order to investigate whether compound **9a** induces caspase-dependent or independent cascade we have also studied the expression of caspase-3. Caspase 3 is activated by procaspase 3, therefore the pro form and the active form of caspase 3 constitute a useful biomarker to confirm apoptosis. The results of the current study revealed that compound **9a** decreased the level of procaspase 3 and up-regulated the expression of caspase-3 in MCF-7 cells treated for 24 h with 2 μM **9a** (Figure 5D).To assess the alterations of apoptosis-related death receptor protein levels in MCF-7 cells, the expression of FAS, procaspase-8 and caspase-8 was evaluated by western blot analysis.

As shown in Figure 5E, there was no significant change in the expression of these proteins in treated and untreated groups.

##### The Effect of **9a** on the Expression of Angiogenesis-Related Genes

**Figure 6:**
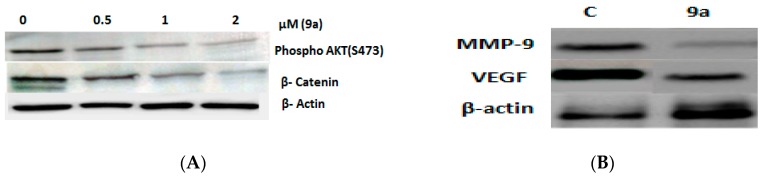
Compound **9a** downregulated metastatic-related genes. (**A**) Compound **9a** down-regulated phosphoAkt and β catenin. Cells were treated with 0.5, 1 and 2 μM of **9a** for 24 h. The cell lysates were collected and the expression of phospho Akt and β catenin was studied by western blot analysis using specific antibodies in treated and non-treated control cells. β-actin was used as a loading control; (**B**) Compound **9a** down-regulated MMP 9 and VEGF. Cells were treated with 2 μM of **9a** for 24 h. The cell lysates were collected and the expression of MMP 9 and VEGF was studied by western blot analysis using specific antibodies in treated and non-treated control cells. β-actin was used as a loading control. C: control (untreated cells).

AKT is a serine/threonine protein kinase that plays a key role in multiple cellular processes including metabolism, proliferation, apoptosis/survival, and migration. Phosphorylation of Akt at S473 activates Akt which is involved in the angiogenic process [61]. On the other hand, the WNT/β-catenin signaling pathway plays an important role in carcinogenesis and tumor metastasis, in many cancer types including breast cancer [62]. Therefore, we further investigated the possible effects of compound **9a** on the expression of phospho AKT and β-catenin. Our data clearly revealed that **9a** reduced the expression of phospho AKT and β-catenin in dose-dependent manner (Figure 6A). The angiogenic response is induced by growth factors such as VEGF, basic fibroblast growth factor (bFGF), platelet-derived growth factor (PDGF), and chemokines. Matrix metallopeptidases (MMPs) such as MMP9 plays an important role in extracellular matrix remodeling, angiogenesis and metastasis and its increased expression was observed in a metastatic mammary cancer cell line [63]. In the present study, the data (Figure 6B) revealed that compound **9a** significantly inhibited expression of VEGF and MMP-9 in MCF-7 cells.

## 3. Experimental Section

### 3.1. General Informstion

Melting points were determined in open-glass capillaries using Stuart capillary melting point apparatus (Stuart Scientific Stone, Staffordshire, UK) and are uncorrected. The IR spectra (KBr) were recorded using a Perkin-Elmer 1430 spectrophotometer (Perkin Elmer, Beaconsfield, UK). The ^1^H-NMR and ^13^C-NMR spectra were determined on a Mercury VX-300 MHz spectrometer (Varian, San Diego, CA, USA) using tetramethylsilane (TMS) as internal standard and DMSO-*d*_6_ as solvent (chemical shifts are given in δ ppm). Mass spectra were run on a GCMS-QP 2010 Plus (70 eV) gas chromatograph/mass spectrometer (Shimadzu, Kyoto, Japan). Microanalyses were performed at the Regional Center for Mycology and Biotechnology, El-Azhar University (Cairo, Egypt). The microanalyses results were within ±0.4% of the calculated values. Monitoring of reactions and checking the purity of the compounds was done by thin layer chromatography (TLC) on silica gel-precoated aluminium sheets (Type 60 GF254; Merck, Munich, Germany) and the spots were detected by exposure to UV lamp at *λ* 254 nm for a few seconds.

### 3.2. Chemistry

#### 3.2.1. *4,6-bis(3,4-Dimethoxyphenyl)-2-oxo-1,2-dihydropyridine-3-carbonitrile* (**3**)

A mixture of 3,4-dimethoxybenzaldehyde **1** (1.66 g, 0.01 mol), 3,4-dimethoxyacetophenone **2** (1.8 g, 0.01 mol), ethyl cyanoacetate (1.13 g, 0.01 mol) and anhydrous ammonium acetate (7.71 g, 0.1 mol) in ethanol (50 mL) was stirred and heated under reflux for 12 h. The reaction mixture was allowed to cool and the obtained precipitate was filtered, washed with cold ethanol, dried and crystallized from dimethyl formamide/ethanol (2:1). Yield: 70%, m.p.: 180–182 °C. IR (ν cm^−1^): 3455 (NH), 2216 (CN), 1653 (C=O), 1267, 1021 (C-O-C). ^1^H-NMR (ppm): 3.84 (s, 6H, 2 OCH_3_), 3.87 (s, 6H, 2 OCH_3_), 6.83 (s, 1H, pyridine C_5_-H), 7.09 (d, *J* = 8.7 Hz, 1H, dimethoxyphenyl C_5_-H), 7.14 (d, *J* = 9.0 Hz, 1H, dimethoxyphenyl C_5_-H), 7.33 (s, 1H, dimethoxyphenyl C_2_-H), 7.35 (s, 1H, dimethoxyphenyl C_2_-H), 7.47 (d, *J* = 8.7 Hz, 1H, dimethoxyphenyl C_6_-H), 7.53 (d, *J* = 9.0 Hz, 1H, dimethoxyphenyl C_6_-H), 12.25 (s, 1H, NH). Anal. Calcd (%) for C_22_H_20_N_2_O_5_ (392.4): C, 67.34; H, 5.14; N, 7.14. Found: C, 67.09; H, 5.11; N, 6.99.

#### 3.2.2. General Procedure for the Synthesis of Compounds **4a**–**d**

To a solution of the cyanopyridone **3** (0.39 g, 0.001 mol) in dry acetone (5 mL) containing anhydrous potassium carbonate (0.14 g, 0.001 mol), the appropriate alkyl halide or chloroacetone (0.001 mol) was added dropwise. The reaction mixture was stirred and heated under reflux for 2 h then allowed to attain room temperature. It was then poured onto crushed ice and the separated solid product was filtered, washed with water, dried and crystallized from the proper solvent.

*2-Methoxy-4,6-bis(3,4-dimethoxyphenyl)pyridine-3-carbonitrile* (**4a**). Yield: 72%, m.p.: 155–156 °C (ethanol). IR (ν cm^−1^): 2213 (CN), 1586 (C=N), 1263, 1223, 1024 (C-O-C). ^1^H-NMR (ppm): 3.71 (s, 3H, OCH_3_), 3.73 (s, 3H, OCH_3_), 3.79 (s, 3H, OCH_3_), 3.81 (s, 3H, OCH_3_), 3.83 (s, 3H, OCH_3_), 6.98 (d, *J* = 8.7 Hz, 1H, dimethoxyphenyl C_5_-H), 7.07 (d, *J* = 8.4 Hz, 1H, dimethoxyphenyl C_5_-H), 7.31–7.37 (m, 2H, dimethoxyphenyl C_2,6_-H), 7.75 (s, 1H, pyridine C_5_-H), 7.78 (d, *J* = 2.1 Hz, 1H, dimethoxyphenyl C_2_-H), 7.79 (d, *J* =8.4, 2.1 Hz, 1H, dimethoxyphenyl C_6_-H). ^13^C-NMR (DMSO-*d*_6_, ppm): 55.36, 55.42, 55.56, 55.60, 55.64, 90.46, 110.12, 111.74, 111.79, 111.89, 113.13, 115.69, 120.64, 121.56, 127.83, 128.91, 148.59, 148.81, 150.23, 151.12, 156.10, 156.69, 163.08. Anal. Calcd (%) for C_23_H_22_N_2_O_5_ (406.43): C, 67.97; H, 5.46; N, 6.89. Found: C, 68.13; H, 5.44; N, 7.03.

*2-Ethoxy-4,6-bis(3,4-dimethoxyphenyl)pyridine-3-carbonitrile* (**4b**). Yield: 73%, m.p.: 152–153 °C (ethanol). IR (ν, cm^−1^): 2220 (CN), 1582 (C=N), 1265, 1226, 1027 (C-O-C). ^1^H-NMR (ppm): 1.44 (t, *J* = 6.9 Hz, 3H, CH_3_), 3.84 (s, 3H, OCH_3_), 3.85 (s, 3H, OCH_3_), 3.86 (s, 3H, OCH_3_), 3.87 (s, 3H, OCH_3_), 4.62 (q, *J* = 6.9 Hz, 2H, OCH_2_), 7.10 (d, *J* = 8.9 Hz, 1H, dimethoxyphenyl C_5_-H), 7.15 (d, *J* = 8.3 Hz, 1H, dimethoxyphenyl C_5_-H), 7.29–7.35 (m, 2H, dimethoxyphenyl C_2,6_-H), 7.75 (s, 1H, pyridine C_5_-H), 7.78 (d, *J* = 2.1 Hz, 1H, dimethoxyphenyl C_2_-H), 7.80 (dd, *J* = 8.3, 2.1 Hz, 1H, dimethoxyphenyl C_6_-H). ^13^C-NMR (ppm): 14.8, 55.45, 55.58, 55.59, 55.66, 63.88, 90.49, 110.14, 111.73, 111.77, 111.94, 113.18, 115.72, 120.73, 121.64, 127.94, 129.01, 148.66, 148.84, 150.19, 151.18, 156.21, 156.82, 163.22. EI-MS *m*/*z* (%): 420.17 (M^+^) (77 %), 111 (100%). Anal. Calcd (%) for C_24_H_24_N_2_O_5_ (420.46): C, 68.56; H, 5.75; N, 6.66. Found: C, 68.72; H, 5.78; N, 6.79.

*4,6-bis(3,4-Dimethoxyphenyl)-2-propoxypyridine-3-carbonitrile* (**4c**). Yield: 64%, m.p.: 156–158 °C (dimethylformamide/ethanol 2:1). IR (ν cm^−1^): 2214 (CN), 1583 (C=N), 1264, 1228, 1026 (C-O-C). ^1^H-NMR (ppm): 0.93 (t, *J* = 6.9 Hz, 3H, CH_2_CH_3_), 1.65–1.70 (m, 2H, CH_2_CH_3_), 3.77 (s, 3H, OCH_3_), 3.80 (s, 3H, OCH_3_), 3.82 (s, 3H, OCH_3_), 3.84 (s, 3H, OCH_3_), 4.83 (t, *J* = 6.9 Hz, 2H, OCH_2_), 6.96 (d, *J* = 8.3 Hz, 1H, dimethoxyphenyl C_5_-H), 7.08 (d, *J* = 8.5 Hz, 1H, dimethoxyphenyl C_5_-H), 7.22 (dd, *J* = 8.3, 1.8 Hz, 1H, dimethoxyphenyl C_6_-H), 7.26 (d, *J* = 1.8 Hz, 1H, dimethoxyphenyl C_2_-H), 7.67 (s, 1H, pyridine C_5_-H), 7.70 (d, *J* = 1.8 Hz, 1H, dimethoxyphenyl C_2_-H), 7.77 (dd, *J* = 8.5, 1.8 Hz, 1H, dimethoxyphenyl C_6_-H). ^13^C-NMR (ppm): 11.76, 23.84, 55.49, 55.55, 55.61, 55.68, 65.23, 90.56, 110.22, 111.65, 111.82, 112.12, 113.33, 115.68, 120.80, 121.66, 128.01, 129.14, 148.73, 148.69, 150.26, 151.34, 156.27, 156.94, 163.25. Anal. Calcd (%) for C_25_H_26_N_2_O_5_ (434.48): C, 69.11; H, 6.03; N, 6.45. Found: C, 68.77; H, 5.73; N, 6.59.

*4,6-bis(3,4-Dimethoxyphenyl)-2-(2-oxopropoxy)pyridine-3-carbonitrile* (**4d**). Yield: 75%, m.p.: 110–112 °C (ethanol). IR (ν cm^−1^): 2214 (CN), 1731 (C=O), 1593 (C=N), 1262, 1023 (C-O-C). ^1^H-NMR (ppm): 2.23 (s, 3H, COCH_3_), 3.72 (s, 3H, OCH_3_), 3.78 (s, 3H, OCH_3_), 3.80 (s, 3H, OCH_3_), 3.83 (s, 3H, OCH_3_), 5.25 (s, 2H, OCH_2_), 7.08 (d, *J* = 8.7 Hz, 1H, dimethoxyphenyl C_5_-H), 7.17 (d, *J* = 8.4 Hz, 1H, dimethoxyphenyl C_5_-H), 7.33 (dd, *J* = 8.7 Hz, 2.1 Hz, 1H, dimethoxyphenyl C_6_-H), 7.37 (d, *J* = 2.1 Hz, 1H, dimethoxyphenyl C_2_-H), 7.66 (s, 1H, pyridine C_5_-H), 7.77 (d, *J* = 8.4 Hz, 1H, dimethoxyphenyl C_6_-H), 7.78 (d, *J* = 2.1 Hz, 1H, dimethoxyphenyl C_2_-H). ^13^C-NMR (ppm):25.73, 55.47, 55.54, 55.57, 55.62, 70.62, 90.42, 110.07, 111.63, 111.71, 111.92, 113.05, 115.66, 120.75, 121.50, 127.87, 128.84, 148.54, 148.76, 150.29, 151.06, 156.07, 156.64, 162.96, 203.32. Anal. Calcd (%) for C_25_H_24_N_2_O_6_ (448.47): C, 66.95; H, 5.39; N, 6.25. Found: C, 67.15; H, 5.44; N, 6.21.

#### 3.2.3. *Ethyl 2-(3-Cyano-4,6-bis(3,4-dimethoxyphenyl)pyridin-2-yloxy)acetate* (**5**)

A mixture of **3** (3.92 g, 0.01 mol), ethyl bromoacetate (1.67 g, 0.01 mol) and anhydrous potassium carbonate (5.53 g, 0.04 mol) in dry acetone (30 mL) was heated under reflux for 20 h. After cooling, water was added and the reaction mixture was left in a refrigerator for an overnight. The separated solid product was filtered, washed with water, dried and recrystallized from ethanol. Yield: 72%, m.p.: 130–132 °C. IR (ν cm^−1^): 2220 (CN), 1760 (C=O), 1595 (C=N), 1258, 1231, 1067, 1028 (C-O-C). ^1^H-NMR (ppm): 1.15 (t, *J* = 7.1 Hz, 3H, CH_2_CH_3_), 3.84 (s, 3H, OCH_3_), 3.85 (s, 3H, OCH_3_), 3.86 (s, 6H, 2OCH_3_), 4.17 (q, *J* = 7.1 Hz, 2H, CH_2_CH_3_), 5.14 (s, 2H, OCH_2_), 7.08 (d, *J* = 8.4 Hz, 1H, dimethoxyphenyl C_5_-H), 7.16 (d, *J* = 8.3 Hz, 1H, dimethoxyphenyl C_5_-H), 7.32–7.38 (m, 2H, dimethoxyphenyl C_2,6_-H), 7.73 (d, *J* = 1.9 Hz, 1H, dimethoxyphenyl C_2_-H), 7.81 (dd, *J* = 8.3, 1.9 Hz, 1H, C_6_-H), 7.84 (s, 1H, pyridine C_5_-H). ^13^C-NMR (ppm):13.88, 55.51, 55.63, 55.59, 55.63, 60.81, 63.61, 90.45, 110.34, 111.57, 111.72, 111.99, 113.12, 115.52, 120.79, 121.53, 127.82, 128.66, 148.58, 148.78, 150.35, 151.22, 156.21, 156.47, 162.82, 168.51. Anal. Calcd (%) for C_26_H_26_N_2_O_7_ (478.49): C, 65.26; H, 5.48; N, 5.85. Found: C, 65.38; H, 5.53; N, 5.97.

#### 3.2.4. *2-{[3-Cyano-4,6-bis(3,4-dimethoxyphenyl)pyridin-2-yl]oxy}acetohydrazide* (**6**)

To a suspension of **5** (4.8 g, 0.01 mol) in absolute ethanol (50 mL), hydrazine hydrate 98% (3.2 g, 0.1 mol) was added. The reaction mixture was heated under reflux for 8 h then allowed to cool to room temperature. The obtained product was filtered, washed with ethanol, dried and recrystallized from ethyl alcohol/dimethylformamide mixture (3:1). Yield: 68%, m.p.: 216–218°C. IR (ν cm^−1^): 3437, 3303 (NH), 2209 (CN), 1690 (C=O), 1645 (C=N), 1268, 1019 (C-O-C). ^1^H-NMR (ppm): 3.83 (s, 3H, OCH_3_), 3.85 (s, 3H, OCH_3_), 3.86 (s, 3H, OCH_3_), 3.88 (s, 3H, OCH_3_), 4.27 (s, 2H, NH_2_, D_2_O exchangeable), 4.97 (s, 2H, OCH_2_), 7.07 (d, *J* = 8.7 Hz, 1H, dimethoxyphenyl C_5_-H), 7.17 (d, *J* = 8.8 Hz, 1H, dimethoxy-phenyl C_5_-H), 7.29–7.35 (m, 2H, dimethoxyphenyl C_2,6_-H), 7.75 (d, *J* = 2.0 Hz, 1H, dimethoxyphenyl C_2_-H), 7.80 (s, 1H, pyridine C_5_-H), 7.82 (dd, *J* = 8.8, 2.0 Hz, 1H, dimethoxyphenyl C_6_-H), 9.36 (s, 1H, NH). ^13^C-NMR (ppm): 55.54, 55.66, 55.56, 55.67, 63.65, 90.44, 110.38, 111.63, 111.70, 112.22, 113.16, 115.56, 120.77, 121.55, 127.88, 128.74, 148.68, 148.82, 150.42, 151.26, 156.18, 156.51, 162.85, 168.46. Anal. Calcd (%) for C_24_H_24_N_4_O_6_ (464.47): C, 62.06; H, 5.21; N, 12.06. Found: C, 62.29; H, 5.28; N, 12.12.

#### 3.2.5. General Procedure for the Synthesis of Compounds **7a**–**d**

To a well stirred cold suspension of fine powdered potassium hydroxide (0.11 g, 0.002 mol) in dimethylformamide (4 mL) the acid hydrazide **6** (0.46 g, 0.001 mol) was added followed by carbon disulphide (0.076 g, 0.001 mol). The reaction mixture was stirred at room temperature overnight then the appropriate alkyl halide (0.001 mol) was added and the mixture was further stirred at room temperature for 12 h. The reaction mixture was concentrated under reduced pressure then poured into ice-cold water and the obtained precipitate was filtered, washed with water, dried and crystallized from ethanol.

*Methyl 2-[2-(3-cyano-4,6-bis(3,4-dimethoxyphenyl)pyridin-2-yloxy)acetyl]hydrazinecarbodithioate* (**7a**). Yield: 63%, m.p.:151–153 °C. IR (ν cm^−1^): 3433 (NH), 2214 (CN), 1675 (C=O), 1589 (C=N), 1516, 1314, 1145, 977 (NCS), 1261, 1023 (C-O-C). ^1^H-NMR (ppm): 2.69 (s, 3H, SCH_3_), 3.84 (s, 3H, OCH_3_), 3.85 (s, 3H, OCH_3_), 3.86 (s, 3H, OCH_3_), 3.91 (s, 3H, OCH_3_), 5.78 (s, 2H, OCH_2_), 7.07 (d, *J* = 8.7 Hz, 1H, dimethoxyphenyl C_5_-H), 7.16 (d, *J* = 8.4 Hz, 1H, dimethoxyphenyl C_5_-H), 7.32–7.38 (m, 2H, dimethoxyphenyl C_2,6_-H), 7.80 (d, *J* = 1.8 Hz, 1H, dimethoxyphenyl C_2_-H), 7.84–7.88 (m, 2H, dimethoxyphenyl C_6_-H and pyridine C_5_-H), 10.40 (s, 1H, NH), 10.59 (s, 1H, NH). ^13^C-NMR (ppm):16.28, 55.67, 58.14, 64.21, 91.25, 110.37, 111.59, 111.77, 112.22, 113.62, 115.39, 120.84, 121.56, 127.85, 128.72, 148.63, 148.90, 150.42, 151.31, 156.29, 156.74, 162.44, 164.60, 172.26. EI-MS *m*/*z* (%): 554 (M^+^) (19.5 %), 94 (100%). Anal. Calcd (%) for C_26_H_26_N_4_O_6_S_2_ (554.64): C, 56.30; H, 4.72; N, 10.10. Found: 56.38; H, 4.75; N, 10.21.

*Ethyl 2-[2-(3-cyano-4,6-bis(3,4-dimethoxyphenyl)pyridin-2-yloxy)acetyl]hydrazinecarbodithioate* (**7b**). Yield: 64%, m.p.: 144–146 °C. IR (ν cm^−1^): 3437 (NH), 2214 (CN), 1672 (C=O), 1591 (C=N), 1516, 1307, 1144, 979 (NCS), 1261, 1025 (C-O-C). ^1^H-NMR (ppm): 1.28 (t, *J* = 7.1 Hz, 3H, CH_3_), 3.18 (q, *J* = 7.1 Hz, 2H, SCH_2_), 3.76 (s, 3H, OCH_3_), 3.82 (s, 3H, OCH_3_), 3.83 (s, 3H, OCH_3_), 3.88 (s, 3H, OCH_3_), 5.83 (s, 2H, OCH_2_), 7.07 (d, *J* = 9.0 Hz, 1H, dimethoxyphenyl C_5_-H), 7.16 (d, *J* = 8.1 Hz, 1H, dimethoxyphenyl C_5_-H), 7.28-7.32 (m, 2H, dimethoxyphenyl C_2,6_-H), 7.73–7.86 (m, 3H, dimethoxyphenyl C_2,6_-H and pyridine C_5_-H), 10.42 (s, 1H, NH), 10.63 (s, 1H, NH). ^13^C-NMR (ppm): 14.54, 26.61, 55.64, 58.07, 63.98, 90.78, 110.49, 111.56, 111.74, 112.14, 113.58, 115.37, 120.87, 121.54, 127.80, 128.68, 148.61, 148.89, 150.40, 151.24, 156.33, 156.70, 162.41, 163.78, 171.98. Anal. Calcd (%) for C_27_H_28_N_4_O_6_S_2_ (568.66): C, 57.03; H, 4.96; N, 9.85. Found: C, 57.17; H, 4.92; N, 9.91.

*2-Morpholinoethyl-2-[2-(3-cyano-4,6-bis(3,4-dimethoxyphenyl)pyridin-2-yloxy)acetyl]hydrazinecarbo-dithioate* (**7c**). Yield: 66 %, m.p.: 158–160 °C. IR (ν, cm^−1^): 3435 (NH), 2217 (CN), 1656 (C=O), 1586 (C=N), 1516, 1322, 1138, 921 (NCS), 1262, 1022 (C-O-C). ^1^H-NMR (ppm): 2.60–2.64 (m, 4H, morpholine C_3,5_-H_2_), 2.74 (t, *J* = 6.9 Hz, 2H, SCH_2_), 2.94 (t, *J* = 6.9 Hz, 2H, NCH_2_), 3.59–3.64 (m, 4H, morpholine C_2,6_-H_2_), 3.78 (s, 3H, OCH_3_), 3.83 (s, 3H, OCH_3_), 3.84 (s, 3H, OCH_3_), 3.88 (s, 3H, OCH_3_), 5.72 (s, 2H, OCH_2_), 7.06 (d, *J* = 8.1 Hz, 1H, dimethoxyphenyl C_5_-H), 7.14 (d, *J* = 8.4 Hz, 1H, dimethoxyphenyl C_5_-H), 7.28–7.33 (m, 2H, dimethoxyphenyl C_2,6_-H), 7.75–7.81 (m, 3H, dimethoxyphenyl C_2,6_-H and pyridine C_5_-H), 10.49 (s, 1H, NH), 10.67 (s, 1H, NH). ^13^C-NMR (ppm): 32.83, 52.10, 55.65, 55.67, 56.13, 58.07, 64.69, 90.73, 110.41, 111.57, 111.72, 113.11, 113.58, 115.36, 120.86, 121.55, 127.80, 128.67, 148.59, 148.89, 150.38, 151.21, 156.37, 156.72, 162.35, 164.30, 172.0. Anal. Calcd (%) for C_31_H_35_N_5_O_7_S_2_ (653.77): C, 56.95; H, 5.40; N, 10.71. Found: 56.61; H, 5.18; N, 11.04.

*2-(Piperidin-1-yl)-ethyl-2-[2-(3-cyano-4,6-bis(3,4-dimethoxyphenyl)pyridin-2-yloxy)acetyl]hydrazinecarbo-dithioate* (**7d**). Yield: 66 %, m.p.: 160–162 °C. IR (ν, cm^−1^): 3434 (NH), 2218 (CN), 1645 (C=O), 1587 (C=N), 1516, 1322, 1134, 921 (NCS), 1262, 1022 (C-O-C). ^1^H-NMR (ppm): 1.44–1.48 (m, 2H, piperidine C_4_-H_2_), 1.60–1.64 (m, 4H, piperidine C_3,5_-H_2_), 2.83–3.07 (m, 8H, piperidine C_2,6_-H, SCH_2_ and NCH_2_), 3.86 (s, 3H, OCH_3_), 3.87 (s, 3H, OCH_3_), 3.90 (s, 3H, OCH_3_), 3.92 (s, 3H, OCH_3_), 5.64 (s, 2H, OCH_2_), 7.08 (d, *J* = 8.1 Hz, 1H, dimethoxyphenyl C_5_-H), 7.20 (d, *J* = 8.4 Hz, 1H, dimethoxyphenyl C_5_-H), 7.33–7.38 (m, 2H, dimethoxyphenyl C_2,6_-H), 7.71–7.85 (m, 3H, dimethoxyphenyl C_2,6_-H and pyridine C_5_-H), 10.51 (s, 1H, NH), 10.69 (s, 1H, NH). ^13^C-NMR (ppm): 23.76, 33.14, 52.26, 53.82, 55.65, 55.67, 63.99, 91.33, 110.42, 111.55, 111.76, 113.14, 113.59, 115.54, 120.88, 121.61, 127.84, 128.69, 148.55, 148.93, 150.27, 151.33, 156.44, 156.81, 162.46, 164.42, 174.20. Anal. Calcd (%) for C_32_H_37_N_5_O_6_S_2_ (651.8): C, 58.97; H, 5.72; N, 10.74. Found: 58.99; H, 5.83; N, 10.92.

#### 3.2.6. General Procedure for the Synthesis of Compounds **8a**–**c**

A mixture of **6** (0.464 g, 0.001 mol) and the appropriate aldehyde (0.001 mol) in absolute ethanol (20 mL) containing 2 drops of glacial acetic acid was heated under reflux for 8 h. The reaction mixture was allowed to attain room temperature and the separated solid product was filtered, washed with ethanol, dried and recrystallized from dimethylformamide.

*2-[3-Cyano-4,6-bis(3,4-dimethoxyphenyl)pyridin-2-yl]oxy-N'-(4-methoxybenzylidene)acetohydrazide* (**8a**). Yield: 84%, m.p.: 163–164 °C. IR (ν, cm^−1^): 3189 (NH), 2224 (CN), 1673 (C=O), 1601 (C=N), 1257, 1026 (C-O-C). ^1^H-NMR (ppm):3.64 (s, 3H, OCH_3_), 3.78 (s, 3H, OCH_3_), 3.80 (s, 3H, OCH_3_), 3.86 (s, 3H, OCH_3_), 3.88 (s, 3H, OCH_3_), 5.09 (s, 2H, OCH_2_, *cis* conformer), 5.60 (s, 2H, OCH_2_, *trans* conformer), 6.95–7.03 (m, 3H, dimethoxyphenyl C_5_-H and methoxyphenyl C_3,5_-H), 7.18 (d, *J* = 8.4 Hz, 1H, dimethoxyphenyl C_5_-H), 7.33–7.37 (m, 2H, dimethoxyphenyl C_2,6_-H), 7.62–7.72 (m, 4H, dimethoxyphenyl C_2,6_-H and methoxyphenyl C_2,6_-H), 7.79 (s, 1H, pyridine C_5_-H), 8.0 (s, 1H, N=CH, *cis* conformer), 8.22 (s, 1H, N=CH, *trans* conformer), 11.58 (s, 1H, NH, *cis* conformer), 11.62 (s, 1H, NH, *trans* conformer). ^13^C-NMR (ppm): 55.22, 55.51, 55.64, 70.15, 90.45, 109.94, 111.50, 111.76, 112.30, 113.59, 114.29, 115.66, 120.73, 121.48, 127.40, 128.10, 128.33, 128.43, 142.50, 148.55, 148.85, 150.38, 151.19, 155.80, 156.04, 160.75, 163.50, 164.34. Anal. Calcd (%) for C_32_H_30_N_4_O_7_ (582.6): C, 65.97; H, 5.19; N, 9.62. Found: C, 66.12; H, 5.43; N, 9.91.

*2-[3-Cyano-4,6-bis(3,4-dimethoxyphenyl)pyridin-2-yl]oxy-N'-(3,4-dimethoxybenzylidene)acetohydrazide* (**8b**).Yield: 82%, m.p.:190–192 °C. IR (ν, cm^−1^): 3200, 3133 (NH), 2210 (CN), 1696 (C=O), 1581 (C=N), 1264, 1023 (C-O-C). ^1^H-NMR (DMSO-*d*_6_, ppm): 3.66 (s, 3H, OCH_3_), 3.77 (s, 3H, OCH_3_), 3.80 (s, 3H, OCH_3_), 3.82 (s, 3H, OCH_3_), 3.86 (s, 3H, OCH_3_), 3.87 (s, 3H, OCH_3_), 5.11 (s, 2H, OCH_2_, *cis* conformer), 5.62 (s, 2H, OCH_2_, *trans* conformer), 6.96 (d, *J* = 8.7 Hz, 1H, dimethoxyphenyl C_5_-H), 7.16–7.20 (m, 2H, two dimethoxyphenyl C_5_-H), 7.33–7.79 (m, 6H, three dimethoxyphenyl C_2,6_-H), 7.98 (s, 1H, pyridine C_5_-H), 8.0 (s, 1H, N=CH, *cis* conformer), 8.18 (s, 1H, N=CH, *trans* conformer), 11.68 (s, 1H, NH, *cis* conformer), 11.72 (s, 1H, NH, *trans* conformer). ^13^C-NMR (ppm): 55.20, 55.45, 55.56, 55.62, 70.26, 90.26, 110.10, 110.85, 111.53, 111.77, 111.96, 113.20, 113.83, 115.74, 120.98, 121.82, 123.50, 127.74, 128.44, 129.12, 144.36, 148.58, 148.80, 149.05, 149.85, 150.32, 151.18, 155.93, 156.34, 163.88, 164.42.EI-MS *m*/*z* (%): 612 (M^+^) (14.34%), 69.0 (100%). Anal. Calcd (%) for C_33_H_32_N_4_O_8_ (612.63): C, 64.70; H, 5.26; N, 9.15. Found: C, 64.82; H, 5.22; N, 9.23.

*2-[3-Cyano-4,6-bis(3,4-dimethoxyphenyl)pyridin-2-yl]oxy-N'-(3,4,5-trimethoxybenzylidene)acetohydrazide* (**8c**). Yield: 79 %, m.p.: 232–234 °C. IR (ν, cm^−1^): 3204 (NH), 2217 (CN), 1670 (C=O), 1586 (C=N), 1263, 1022 (C-O-C). ^1^H-NMR (ppm): 3.67 (s, 3H, OCH_3_), 3.70 (s, 3H, OCH_3_), 3.78 (s, 3H, OCH_3_), 3.81 (s, 3H, OCH_3_), 3.83 (s, 3H, OCH_3_), 3.86 (s, 3H, OCH_3_), 3.87 (s, 3H, OCH_3_), 5.10 (s, 2H, OCH_2_, *cis* conformer), 5.63 (s, 2H, OCH_2_, *trans* conformer), 6.98 (d, *J* = 8.7 Hz, 1H, dimethoxyphenyl C_5_-H), 7.06 (s, 1H, trimethoxyphenyl-H), 7.18 (d, *J* = 8.1 Hz, 1H, dimethoxyphenyl C_5_-H), 7.32–7.37 (m, 3H, dimethoxyphenyl-C_2,6_-H and trimethoxyphenyl-H), 7.71 (d, *J* = 1.8 Hz, 1H, dimethoxyphenyl C_2_-H), 7.78–7.81 (m, 2H, pyridine C_5_-H and dimethoxyphenyl C_6_-H), 7.96 (s, 1H, N=CH, *cis* conformer), 8.19 (s, 1H, N=CH, *trans* conformer), 11.76 (s, 1H, N=H, *cis* conformer), 11.80 (s, 1H, NH, *trans* conformer). ^13^C-NMR (ppm): 55.53, 55.68, 56.33, 56.46, 60.45, 70.37, 90.48, 104.60, 110.08, 110.79, 111.62, 111.88, 112.47, 115.58, 120.98, 121.82, 127.78, 128.52, 130.37, 139.64, 144.64, 148.58, 149.05, 149.85, 150.32, 153.76, 155.88, 157.11, 164.29, 165.12. Anal. Calcd (%) for C_34_H_34_N_4_O_9_ (642.66): C, 63.54; H, 5.33; N, 8.72. Found: C, 63.59; H, 5.36; N, 8.81.

#### 3.2.7. General Procedure for the Synthesis of Compounds **9a**,**b** and **10a**,**b**

To a suspension of **6** (0.464 g, 0.001 mol) in dry pyridine (5 mL), the appropriate aroyl chloride or sulfonyl chloride (0.001 mol) was slowly added at 0 °C. The reaction mixture was stirred at room temperature overnight then poured into ice-cold water. The obtained precipitate was filtered, washed with water, dried and crystallized from dimethylformamide/ethanol mixture (3:1).

*N'-[2-(3-cyano-4,6-bis(3,4-dimethoxyphenyl)pyridin-2-yloxy)acetyl]benzohydrazide* (**9a**). Yield: 67 %, m.p.: 222–224 °C. IR (ν, cm^−1^): 3416, 3168 (NH), 2216 (CN), 1680 (C=O), 1605 (C=N), 1261, 1025 (C-O-C). ^1^H-NMR (ppm): 3.84 (s, 3H, OCH_3_), 3.86 (s, 3H, OCH_3_), 3.87 (s, 3H, OCH_3_), 3.89 (s, 3H, OCH_3_), 5.19 (s, 2H, OCH_2_), 7.08 (d, *J* = 8.7 Hz, 1H, dimethoxyphenyl C_5_-H), 7.18 (d, *J* = 9.0 Hz, 1H, dimethoxyphenyl C_5_-H), 7.33–7.76 (m, 2H, dimethoxyphenyl C_2,6_-H), 7.46–7.57 (m, 3H, phenyl C_3,4,5_-H), 7.83–7.92 (m, 5H, phenyl C_2,6_-H, dimethoxyphenyl C_2,6_-H and pyridine C_5_-H), 10.41 (s, 1H, NH), 10.50 (s, 1H, NH). ^13^C-NMR (ppm): 55.56, 55.59, 55.64, 55.67, 64.68, 90.23, 110.27, 111.65, 111.72, 112.31, 113.16, 115.66, 120.73, 121.46, 127.67, 127.82, 128.43, 128.79, 132.45, 133.89, 148.75, 148.69, 150.38, 151.42, 156.18, 156.51, 162.85, 166.12, 168.46. EI-MS *m/z* (%): 568.0 (M^+^) (16.36 %), 153.90 (100%). Anal. Calcd (%) for C_31_H_28_N_4_O_7_ (568.58): C, 65.48; H, 4.96; N, 9.85. Found: C, 65.56; H, 4.98; N, 9.97.

*N'-[2-(3-cyano-4,6-bis(3,4-dimethoxyphenyl)pyridin-2-yloxy)acetyl]-4-methoxybenzo hydrazide* (**9b**). Yield: 69%, m.p.: 170–172 °C. IR (ν, cm^−1^): 3440, 3173 (NH), 2218 (CN), 1667 (C=O), 1604 (C=N), 1261, 1023 (C-O-C). ^1^H-NMR (ppm): 3.81 (s, 3H, OCH_3_), 3.84 (s, 3H, OCH_3_), 3.85 (s, 3H, OCH_3_), 3.86 (s, 3H, OCH_3_), 3.88 (s, 3H, OCH_3_),5.16 (s, 2H, OCH_2_), 7.01 (d, *J* = 9.0 Hz, 1H, dimethoxyphenyl C_5_-H), 7.08 (d, *J* = 8.4 Hz, 1H, dimethoxyphenyl C_5_-H), 7.17 (d, *J* = 8.7 , 2.1 Hz, 2H, methoxyphenyl C_3,5_-H), 7.32–7.35 (m 2H, dimethoxyphenyl C_2,6_-H), 7.81–7.90 (m, 5H, dimethoxyphenyl C_2,6_-H, pyridine C_5_-H and methoxyphenyl C_2,6_-H), 10.33 (s, 2H, 2NH). ^13^C-NMR (ppm): 55.59, 55.62, 55.64, 55.68, 55.77, 63.69, 90.17, 110.14, 111.63, 111.74, 112.13, 113.25, 114.23, 115.69, 120.74, 121.56, 125.81, 127.77, 128.43, 128.72, 148.63, 148.79, 150.44, 151.21, 156.43, 156.58, 163.12, 165.40, 165.90, 168.46. Anal. Calcd (%) for C_32_H_30_N_4_O_8_ (598.6): C, 64.21; H, 5.05; N, 9.36. Found: C, 64.37; H, 5.03; N, 9.44.

*N'-[2-(3-cyano-4,6-bis(3,4-dimethoxyphenyl)pyridin-2-yloxy)acety]benzenesulfohydrazide* (**10a**). Yield: 65%, m.p.: 120–122 °C. IR (ν, cm^−1^): 3454, 3233 (NH), 1711 (C=O), 1587 (C=N), 1330, 1165 (SO_2_), 1261, 1021 (C-O-C). ^1^H-NMR (ppm): 3.83 (s, 3H, OCH_3_), 3.85 (s, 3H, OCH_3_), 3.86 (s, 3H, OCH_3_), 3.90 (s, 3H, OCH_3_), 4.96 (s, 2H, OCH_2_), 7.06 (d, *J* = 9.0 Hz, 1H, dimethoxyphenyl C_5_-H), 7.16 (d, *J* = 8.9 Hz, 1H, dimethoxyphenyl C_5_-H), 7.23–7.85 (m, 10H, two dimethoxyphenyl C_2,6_-H, pyridine C_5_-H and phenyl-H), 10.06 (s, 1H, NH), 10.49 (s, 1H, NH). ^13^C-NMR (ppm): 55.48, 55.56, 55.62, 55.68, 64.66, 90.29, 110.25, 111.66, 111.74, 112.36, 113.14, 115.60, 120.69, 121.50, 127.64, 128.08, 128.47, 128.89, 132.39, 138.91, 148.68, 148.73, 150.42, 151.44, 156.22, 156.54, 162.93, 166.48. Anal. Calcd (%) for C_30_H_28_N_4_O_8_S (604.63): C, 59.59; H, 4.67; N, 9.27. Found: C, 59.78; H, 4.60; N, 9.39.

*N'-[2-(3-cyano-4,6-bis(3,4-dimethoxyphenyl)pyridin-2-yloxy)acetyl]-4-methoxybenzenesulfohydrazide* (**10b**). Yield: 65 %, m.p.: 190–192 °C. IR (ν, cm^−1^): 3446, 3238 (NH), 1708 (C=O), 1588 (C=N), 1333, 1166 (SO_2_), 1258, 1030 (C-O-C). ^1^H-NMR (ppm): 3.80 (s, 3H, OCH_3_), 3.82 (s, 3H, OCH_3_), 3.86 (s, 3H, OCH_3_), 3.87 (s, 3H, OCH_3_), 3.89 (s, 3H, OCH_3_), 4.97 (s, 2H, OCH_2_), 7.0 (d, *J* = 9.0 Hz, 1H, dimethoxyphenyl C_5_-H), 7.09 (d, *J* = 8.6 Hz, 1H, dimethoxyphenyl C_5_-H), 7.16 (d, *J* = 8.8 , 2.1 Hz, 2H, methoxyphenyl C_3,5_-H), 7.30–7.34 (m, 2H, dimethoxyphenyl C_2,6_-H), 7.82–7.88 (m, 5H, dimethoxyphenyl C_2,6_-H, pyridine C_5_-H and methoxyphenyl C_2,6_-H), 10.10 (s, 1H, NH), 10.48 (s, 1H, NH). ^13^C-NMR (ppm): 55.47, 55.58, 55.62, 55.65, 55.7), 63.6), 90.2), 110.1), 111.58, 111.76, 112.16, 113.22, 114.27, 115.73, 120.77, 121.63, 127.77, 128.43, 128.72, 130.21, 148.68, 148.81, 150.39, 151.26, 156.38, 156.62, 163.14, 165.44, 166.76. Anal. Calcd (%) for C_31_H_30_N_4_O_9_S (634.66): C, 58.67; H, 4.76; N, 8.83. Found: C, 58.78; H, 4.60; N, 9.19.

#### 3.2.8. General Procedure for the Synthesis of Compounds **11** and **12**

A mixture of **6** (0.464 g, 0.001 mol) and acetylacetone or ethyl acetoacetate (0.001 mol) in absolute ethanol/glacial acetic acid mixture (2:1) (15 mL) was heated under reflux for 8 h then allowed to cool to room temperature. The separated solid product was filtered, thoroughly washed with cold ethanol, dried and recrystallized from dimethylformamide/water mixture (4:1).

*4,6-Bis(3,4-dimethoxyphenyl)-2-(2-(3,5-dimethyl-1H-pyrazol-1-yl)-2-oxoethoxy)pyridine-3-carbonitrile* (**11**). Yield: 67%, m.p.: 161–163 °C. IR (ν, cm^−1^): 2215 (CN), 1705 (C=O), 1591 (C=N), 1255, 1022 (C-O-C). ^1^H-NMR (ppm):2.19 (s, 3H, CH_3_), 2.38 (s, 3H, CH_3_), 3.74 (s, 3H, OCH_3_), 3.76 (s, 3H, OCH_3_), 3.78 (s, 3H, OCH_3_), 3.80 (s, 3H, OCH_3_), 5.15 (s, 2H, OCH_2_), 6.21 (s, 1H, pyrazole C_4_-H), 7.10 (d, *J* = 8.6 Hz, 1H, dimethoxyphenyl C_5_-H), 7.16 (d, *J* = 8.3 Hz, 1H, dimethoxyphenyl C_5_-H), 7.33-7.38 (m, 2H, dimethoxyphenyl C_2,6_-H), 7.73 (d, *J* = 2.2 Hz, 1H, dimethoxyphenyl C_2_-H), 7.79 (dd, *J* = 8.3, 2.2 Hz, 1H, dimethoxyphenyl C_6_-H), 7.85 (s, 1H, pyridine C_5_-H). ^13^C-NMR (ppm):11.23, 12.54, 55.49, 55.64, 55.68, 55.71, 63.72, 90.28, 96.36, 110.41, 111.48, 111.73, 112.28, 113.36, 115.65, 120.76, 121.66, 127.79, 128.92, 142.89, 148.77, 148.85, 150.43, 151.33, 152.53, 156.28, 156.62, 162.89, 168.63. Anal. Calcd (%) for C_29_H_28_N_4_O_6_ (528.56): C, 65.90; H, 5.34; N, 10.60. Found: C, 66.08; H, 5.38; N, 10.81.

*4,6-bis(3,4-Dimethoxyphenyl)-2-(2-(3-methyl-5-oxo-4,5-dihydro-1H-pyrazol-1-yl)-2-oxoethoxy)pyridine-3-carbonitrile* (**12**). Yield: 65 %, m.p.: 189–190 °C. IR (ν, cm^−1^): 2216 (CN), 1708 (C=O), 1593 (C=N), 1261, 1023 (C-O-C). ^1^H-NMR (ppm): 2.49 (s, 3H, CH_3_), 3.67 (s, 2H, pyrazole C_4_-H_2_), 3.76 (s, 3H, OCH_3_), 3.78 (s, 3H, OCH_3_),3.81(s, 3H, OCH_3_), 3.83 (s, 3H, OCH_3_), 5.16 (s, 2H, OCH_2_), 7.10 (d, *J* = 8.7 Hz, 1H, dimethoxyphenyl C_5_-H), 7.17 (d, *J* = 8.3 Hz, 1H, dimethoxyphenyl C_5_-H), 7.33–7.38 (m, 2H, dimethoxyphenyl C_2,6_-H), 7.72 (d, *J* = 2.2 Hz, 1H, dimethoxyphenyl C_2_-H), 7.80 (dd, *J* = 8.3, 2.2 Hz, 1H, dimethoxyphenyl C_6_-H), 7.85 (s, 1H, pyridine C_5_-H). ^13^C-NMR (ppm): 14.95, 55.53, 55.65, 55.69, 55.96, 59.60, 63.68, 90.14, 110.29, 111.38, 111.77, 112.32, 113.44, 115.71, 120.77, 121.69, 127.88, 128.97, 142.98, 148.77, 148.85, 150.43, 151.33, 156.33, 156.67, 162.82, 162.88, 168.63. Anal. Calcd (%) for C_28_H_26_N_4_O_7_ (530.53): C, 63.39; H, 4.94; N, 10.56. Found: C, 63.47; H, 4.98; N, 10.73.

#### 3.2.9. General Procedure for the Synthesis of Compounds **13a**,**b**

A mixture of **6** (0.464 g, 0.001 mol) and methyl 2-cyano-3,3-bis(methylthio)acrylate or [bis (methylthio)methylene]malononitrile (0.001 mol) in dry dimethylformamide (8 mL) was heated under reflux for 5 h. The solvent was removed under vacuum and the residue was treated with ice cold water. The formed precipitate was filtered, washed with ethanol, dried and crystallized from dioxane.

*Methyl 5-amino-1-{[(3-cyano-4,6-bis(3,4-dimethoxyphenyl)pyridin-2-yl)oxy]acetyl}-3-(methylsulfanyl)-1H-pyrazole-4-carboxylate* (**13a**). Yield: 64 %, m.p.: 184–186 °C. IR (ν cm^−1^): 3448, 3323 (NH_2_), 2221 (CN), 1739, 1675 (C=O), 1622 (C=N), 1261, 1019 (C-O-C). ^1^H-NMR (ppm): 2.49 (s, 3H, SCH_3_), 3.72 (s, 3H, OCH_3_), 3.80 (s, 3H, OCH_3_), 3.86 (s, 3H, OCH_3_), 3.87 (s, 3H, OCH_3_), 3.88 (s, 3H, OCH_3_), 5.85 (s, 2H, OCH_2_), 6.99 (d, *J* = 8.7 Hz, 1H, dimethoxyphenyl C_5_-H), 7.18 (d, *J* = 8.4 Hz, 1H, dimethoxyphenyl C_5_-H), 7.34–7.38 (m, 2H, dimethoxyphenyl C_2,6_-H), 7.49 (s, 2H, NH_2_, D_2_O exchangeable), 7.56 (s, 1H, dimethoxyphenyl C_2_-H), 7.71 (d, *J* = 8.4 Hz, 1H, dimethoxyphenyl C_6_-H), 7.84 (s, 1H, pyridine C_5_-H). ^13^C-NMR (ppm): 13.15, 51.28, 55.49, 55.64, 55.68, 55.71, 63.72, 90.28, 96.36, 110.41, 111.48, 111.73, 112.28, 113.36, 115.65, 120.76, 121.66, 127.79, 128.92, 142.89, 148.77, 148.85, 150.43, 151.33, 152.53, 156.28, 156.62, 162.89, 168.63. EI-MS *m/z*: 619.0 (M^+^) (5.14%), 64.0 (100%). Anal. Calcd (%) for C_30_H_29_N_5_O_8_S (619.64): C, 58.15; H, 4.72; N, 11.30. Found: C, 58.32; H, 4.48; N, 11.13.

*2-{2-[5-Amino-4-cyano-3-(methylsulfanyl)-1H-pyrazol-1-yl]-2-oxoethoxy}-4,6-bis(3,4-dimethoxyphenyl)-pyridine-3-carbonitrile* (**13b**). Yield 59 %, m.p. 202–204 °C. IR (ν, cm^−1^): 3397, 3168 (NH), 2215 (CN), 1670 (C=O), 1618 (C=N), 1267, 1020 (C-O-C). ^1^H-NMR (ppm): 2.49 (s, 3H, SCH_3_), 3.80 (s, 3H, OCH_3_), 3.86 (s, 3H, OCH_3_), 3.87 (s, 3H, OCH_3_), 3.89 (s, 3H, OCH_3_), 5.11 (s, 2H, OCH_2_), 6.96 (d, *J* = 8.7 Hz, 1H, dimethoxyphenyl C_5_-H), 7.14–7.20 (m, 3H, dimethoxyphenyl C_5_-H and NH_2_), 7.28–7.36 (m, 2H, dimethoxyphenyl C_2,6_-H), 7.70 (s, 1H, dimethoxyphenyl C_2_-H), 7.75 (s, 1H, pyridine C_5_-H), 7.83 (d, *J* = 8.4 Hz, 1H, dimethoxyphenyl C_6_-H). ^13^C-NMR (ppm):13.26, 55.52, 55.59, 55.64, 55.76, 63.78, 90.26, 91.44, 110.41, 111.55, 111.78, 112.36, 113.39, 113.56, 115.68, 120.84, 121.73, 128.11, 128.96, 143.77, 148.78, 148.89, 150.44, 151.36, 153.59, 156.34, 156.65, 162.84, 168.63. EI-MS *m/z* (%): 619.0 (M^+^) (5.14%), 64.0 (100%). Anal. Calcd (%) for C_29_H_26_N_6_O_6_S (586.62): C, 59.38; H, 4.47; N, 14.33. Found: C, 59.69; H, 4.51; N, 14.68.

*2-((5-Mercapto-1,3,4-oxadiazol-2-yl)methoxy)-4,6-bis(3,4-dimethoxyphenyl)pyridine-3-carbonitrile* (**14**). A mixture of **6** (0.464 g, 0.001 mol), carbon disulfide (0.076 g, 0.001 mol) and potassium hydroxide (0.056 g, 0.001 mol) in ethanol (20 mL) was heated under reflux for 8 h. The reaction mixture was left to cool then poured onto crushed ice and acidified with 4N hydrochloric acid. The separated solid was filtered, washed with water, dried and crystalized from dioxane. Yield: 62%, m.p. 180–182 °C. IR (ν, cm^−1^): 3219 (NH), 2218 (CN), 1629 (C=N), 1262,1022(C-O-C). ^1^H-NMR (ppm): 3.80 (s, 3H, OCH_3_), 3.81 (s, 3H, OCH_3_), 3.82 (s, 3H, OCH_3_), 3.86 (s, 3H, OCH_3_), 5.69 (s, 2H, OCH_2_), 7.03 (d, *J* = 8.9 Hz, 1H, dimethoxyphenyl C_5_-H), 7.12 (d, *J* = 8.6 Hz, 1H, dimethoxyphenyl C_5_-H), 7.29–7.80 (m, 4H, two dimethoxyphenyl C_2,6_-H), 7.82 (s, 1H, pyridine C_5_-H), 12.53 (s, 1H, NH). ^13^C-NMR (ppm): 55.64, 55.69, 55.73, 55.76, 63.88, 90.33, 110.43, 111.54, 111.80, 112.41, 113.42, 115.70, 120.87, 121.75, 128.13, 129.11, 148.81, 148.84, 150.39, 151.45, 158.35, 156.29, 156.71, 162.88,178.30.EI-MS *m/z*: 507.0 (M^+^ /11.40%), 149 (100%). Anal. Calcd (%) for C_25_H_22_N_4_O_6_S (506.53): C, 59.28; H, 4.38; N, 11.06. Found: 59.39; H, 4.35; N, 11.22. 

### 3.3. Biological Evaluation Methodology

#### 3.3.1. Cell Lines and Cell Culture

The following cell lines were used for the screening stage, obtained from the American Type Culture Collection (ATCC, Manassas, VA, USA); SK-OV-3 (HTB-77), MCF-12A (ATCC CRL-10782), MDA-MB-231 (ATCC HTB-26), HCT-116 (CCL-247), RKO (CRL-2577) and sub-culturing of the above cell lines was conducted in 75 cm^2^ flasks. The old culture media was removed and discarded after assessing the confluency of the cells and the cells were rinsed with 2–3 mL diluted trypsin (1×) (trypsin/EDTA solution consists of 0.025% trypsin and 0.01% EDTA in PBS), for about 5 minutes for complete detachment. The cells were observed under the inverted microscope until the cell layer is completely dispersed. 75 cm^2^ flasks were prepared and 16 mL of DMEM media for SK-OV-3, MCF-7, MDA-MB-231, (DMEM 4500 mg glucose) and RPMI media for HCT-116 were added to each flask. The cells were aspirated gently and added to the flasks. DMEM 4500 mg/L high glucose with 10% FBS, 5% streptomycin and penicillin, RPMI 1640 (prepared by adding 5% heat-inactivated FCS and 2 mm glutamine) were used. DMEM was also used for MCF-12A cells with the addition of human epidermal growth factor 20 ng/mL, insulin 0.01 mg/mL and hydrocortisone 500 ng/mL. 

#### 3.3.2. Drug Stock Preparation

The synthesized compounds were screened for their anti-proliferative activity using 5-FU (F6627-1G, Sigma-Aldrich, St. Louis, MO, USA) as reference compound. A concentration of 0.4% DMSO was used in all experiment after ensuring 100% viability of the cell lines with this concentration. A 25 mM stock concentration of all compounds was prepared for further studies and analysis.

#### 3.3.3. Cell Viability Assay (WST-1 Assay)

Cells were trypsinized and placed in a 15 mL Falcon tube, the cells were centrifuged for 3 min at 1000 rpm. Trypsin is decanted using a sterile pipette and then the pellet is loosened by flicking the tube few times then 5–10 mL warm DMEM or RPMI were added to the Falcon tube and the pellet is resuspended. Cell count was done next by taking 10 μL of the suspension into the cell counting slide and read under 10× cells/mL. A cell stock of 50,000 cells/mL was prepared for the 96 wells. Cells (100 μL) were added to each well using a sterile pipette each time to ensure consistency. The experiment was planned ahead of time using excel sheet, 3–6 wells were reserved as blank media while 3–6 wells for vehicle (DMSO). The plate was incubated for 24 h for compound treatment step. DMEM media was used to prepare different dilutions of the compounds. The old DMEM media was removed from the wells using sterile pipette in the hood and the media was replaced with 100 μL media with each compound. The plate was incubated for 24 h. After 24 h incubation, WST-1 reagent (Sigma) was added to each well and then the plate was incubated for 1 h before measuring the absorbance using VICTOR™ Multilabel Plate Reader (Perkin Elmer, Beaconsfield, UK). The absorbance of the colored solution can be quantified by measuring at a certain wavelength (usually between 500–600 nm), in this experiment the first reading was conducted at 540 nm and the second reading at 690 nm for the reference wavelength. The percentage of cell viability was determined as the ratio of the absorbance of the sample *vs* the control. Three independent experiments were performed. The IC_50_ of each derivative was calculated as the concentration showing 50% cell growth inhibition during 24 h of compound treatment. 5-Florouracil (Sigma-Aldrich, F6627-1G) was used as reference standard.

#### 3.3.4. Morphological Examination

Morphological examination was performed as described previously; MCF-7 cells were plated on 96 well plates using 5 × 10^4^ cells/well and allowed to reach 75%–85% confluence. The compounds were added to the medium, untreated cells were used as negative control, in addition to blank cells (medium) and vehicle which consist of (DMSO 0.4% + cells). After treatment, all the cells were incubated for 24 h in an atmosphere containing 5% CO_2_. After 2 h of treatment, the morphological changes of the cells were examined and photographs were taken using an inverted microscope at 400× magnification.

#### 3.3.5. TUNEL Assay

For *in situ* detection of apoptotic cells, a terminal-deoxynucleotidyl transferase meditated nick end labeling (TUNEL) assay was performed using DeadEndTM fluorimetric tunnel system (Promega, Madison, WI, USA). Cells were cultured on 4-chamber slides (VWR, Radnor, PA, USA) at a density of 2 × 10^4^ cells/ chamber. After treatment with compound **9a**, cells were washed with phosphate buffer saline (PBS) and fixed by incubation in 4% paraformaldehyde (PFA) for 20 min at 4 °C, then permeabilized with 0.05% Triton X-100 for 5 min at 4 °C. The fixed cells were then incubated with digoxigenin-conjugated dUTP in terminal deoxynucleotide transferase recombinant (rTdT)-catalyzed reaction and nucleotide mixture for 60 min at 37 °C in a humidified atmosphere and then immersed in stop/wash buffer for 15 min at room temperature. Cells were then washed with PBS to remove unincorporated fluorescein-12-dUTP. After washing with PBS, cells were incubated in 1 μg/mL 2-(4-amidinophenyl)-6-indole carbamidine dihydrochloride (DAPI) and fluorescein isothiocyanate (FITC) solution for 15 min in dark. Cells were observed with fluorescence microscopy (RT slider Spot, Diagnostic Instruments, Inc., Sterling Heights, MI, USA) and photographed at 100× magnification.

#### 3.3.6. Enzyme Linked Immunosorbent Apoptosis Assay

Cells were seeded at a density of 2 × 10^4^/ well in a 96- well plate and incubated for 24 h. Media were changed to media containing compound **9a** (2 μM). Cells were then incubated for 24 h. An ELISA assay was performed using Cell Death Detection ELISA PLUS kit (Roche-Applied Science, Indianapolis, IN, USA) for the quantitative *in vitro* determination of cytoplasmic histone-associated DNA fragments (mono- and oligonucleosomes) in apoptosing cells. Briefly, cells were lysed with 200-μL lysis buffer for 30 min at room temperature. The lysate was centrifuged at 200 g for 10 min. 150 μL of supernatant was collected, of which 20 μL was incubated with anti-histone biotin and anti-DNA peroxidase at room temperature for 2 h. After washing with incubation buffer three times, 100 μL of substrate solution (2,2′-azino-di(3-ethylbenzthiazolinsulphonic acid) was added to each well and incubated for 15–20 min at room temperature. The absorbance was measured using an ELISA reader (Spectra Max Plus) at 405 nm. The control group was cells without compounds treatment. Each assay was done in triplicate and the standard deviation was determined.

#### 3.3.7. Flow Cytometry

##### Cell Cycle Analysis

MCF-7 cells were seeded in 25 cm^2^ flasks until reaching the desirable confluence of 1 million cells and media was changed to media containing compound 9a (2 μM). After 24 h incubation, the cells were harvested by trypsinization to detach the cells without affecting the integrity of the cell membrane. The cells were incubated with PI for 5 min at room temperature, washed twice with PBS and fixed with ice-cold 70% ethanol while vortexing. The cells were then washed and re-suspended in PBS with 5 g/mL RNase A (Sigma) and 50 g/mL PI (Sigma) for analysis by flow cytometry. Cell cycle analysis was performed using FACScan Flow Cytometer (Becton Dickson, Franklin Lakes, NJ, USA) according to the manufacturer’s protocol.

#### 3.3.8. Western Blot

Total protein was extracted from treated and untreated cells using lysis buffer (10 mM Tris-HCl (pH 7.5), 1 mM EDTA, 1% Triton X-100, 150 mM NaCl, 1 mM dithiothretol, 10% glycerol, 0.2 mM phenylmethysulphonyl fluoride and protease inhibitors) for 30–50 min on ice. The extracts were centrifuged at 13,000× *g* for 15 min at 4 °C to remove cell debris. Folin Lowry (Pierce, Grand Island, NY, USA) protein assay was used to figure the protein concentration in the cell lysates. Proteins were resolved by electrophoresis on 8%–10% sodium dodecyl sulphate-polycrylamide gel loading equal amount of proteins per lane. The resolved proteins were transferred onto polyvinylidene difluoride (PVDF) membrane and then probed with primary antibody against the protein of interest prepared in 5% milk/PBS-T. The membrane was washed using phosphate buffer saline with Tween 20 (PBS-T) and then appropriate secondary antibody conjugated to horseradish peroxidase (HRP) was used for visualization of the bands using ECL chemiluminescence kit (GE, Fairfield, CT, USA). Anti-p27, anti-CDK2, anti-CDK4, anti-(p21), anti-(p53), anti-Bax, anti-Akt, anti-Bcl-2, anti-MDM2, anti-cytochrome C, anti-caspase-8, anti-procaspase-8, anti-FAS, anti-VEGF, anti-MMP-9, anti-β-catenin were purchased from SantaCruz (Dallas, TX, USA). Pixel density of the proteins studied was calculated using Image J, version 1.41o, NIH (Bethesda, MD, USA). The values obtained were first normalized to loading control β-actin and the fold change was measured by normalizing to that of the control (0 h) value. At least two independent experiments were performed.

#### 3.3.9. Statistical Analysis

The quantitative ratios of different groups were compared using Student’s *t*-test. Probability values of *p* < 0.05 were regarded as statistically significant. All statistical tests were two sided.

## 4. Conclusions 

In the present study, new 3-cyano-4,6-bis (3,4-dimethoxyphenyl)-2-substituted pyridines were synthesized and investigated for their cytotoxicity on five cancer cell lines. The results demonstrated that the benzohydrazide derivative **9a** reduced viability and induced apoptosis in MCF 7 breast cancer cells at an IC_50_ value of 2 μM and was less cytotoxic to normal breast epithelial cells (MCF-12a) than MCF 7 cells. The results of apoptosis assays and cell cycle analysis demonstrated that compound **9a** obviously inhibits the proliferation of MCF-7 cancer cells by inducing apoptosis and arresting the cell cycle at G1 phase via inhibition of CDK2 and CDK4. In addition, **9a** modified apoptotic response and increased the expression levels of p53, p21, p27, Bax and caspase-3, while it reduced expression levels of Bcl-2, Mdm-2 and Akt. Additionally, **9a** induced the release of cytochrome c from mitochondria to cytoplasm while it has no significant effect on death-receptor related proteins FAS, procaspase-8 and caspase-8. Collectively, these results suggest that intrinsic (mitochondria) dependent pathways contributed to **9a**-provoked apoptotic death in MCF-7 cells. Moreover, the expression of β-catenin and phospho AKT was downregulated by **9a**. Additionally, compound **9a** was found to significantly inhibit the expression of MMP-9 and VEGF. Finally, the observed data suggest that the 3-cyano-2-substituted alkoxypyridine moiety might be a potential scaffold for further development of more potent anticancer agents and compound **9a** might be a promising novel therapeutic agent with anti-metastatic activities in breast cancer.

## Figures and Tables

**Table 1:** IC_50_ (μM) ^a^ of the synthesized 3-cyano-2-substituted pyridines against five cancer cell lines.

Compound No.	SK-OV-3 ^b^	MCF-7 ^c^	MDA-MB-231 ^d^	HCT-116 ^e^	RKO ^f^
**4a**	11.37 ± 0.72	9.08 ± 0.37	32.10 ± 0.84	20.30 ± 0.85	13.21 ± 0.77
**4b**	18.16 ± 0.98	15.52 ± 0.46	40.04 ± 1.36	26.25 ± 1.22	19.80 ± 0.37
**4c**	22.12 ± 1.32	12.28 ± 0.77	44.93 ± 1.74	10.06 ± 0.76	18.14 ± 1.03
**4d**	25.19 ± 0.76	20.30 ± 0.98	35.80 ± 0.96	13.14 ± 0.84	17.39 ± 0.44
**5**	10.15 ± 0.44	15.42 ± 0.94	25.12 ± 0.87	14.88 ± 0.98	22.13 ± 0.97
**7a**	9.01 ± 0.42	11.23 ± 0.22	30.10 ± 0.88	23.20 ± 0.95	11.25 ± 0.91
**7b**	12.33 ± 0.88	18.14 ± 1.76	37.87 ± 1.25	9.10 ± 0.97	15.21 ± 1.13
**7c**	5.12 ± 0.11	7.10 ± 0.44	31.73 ± 0.89	26.90 ± 0.97	20.30 ± 0.76
**7d**	10.10 ± 0.98	14.30 ± 0.95	35.53 ± 1.03	11.16 ± 1.26	24.98 ± 1.33
**8a**	14.13 ± 0.95	12.01 ± 0.96	25.34 ± 1.24	28.82 ± 0.31	12.32 ± 0.02
**8b**	10.21 ± 0.92	9.31 ± 0.98	32.26 ± 0.87	25.20 ± 0.89	14.16 ± 0.91
**8c**	12.14 ± 0.98	15.27 ± 0.74	20.31 ± 1.06	25.87 ± 0.83	16.32 ± 0.37
**9a**	9.06 ± 0.24	2.04 ± 0.13	22.34 ± 0.88	7.12 ± 0.37	8.22 ± 0.99
**9b**	7.08 ± 0.16	10.18 ± 0.92	19.27 ± 0.86	17.90 ± 0.74	9.95 ± 1.06
**10a**	11.34 ± 0.02	13.39 ± 0.98	26.82 ± 0.33	21.82 ± 0.85	12.10 ± 1.26
**10b**	9.17 ± 0.38	8.12 ± 0.47	22.44 ± 1.71	16.09 ± 1.07	15.65 ± 0.86
**11**	26.19 ± 0.95	>50	>50	33.90 ± 0.87	>50
**12**	20.27 ± 1.72	>50	>50	40.35 ± 1.76	>50
**13a**	>50	>50	>50	36.30 ± 1.71	>50
**13b**	>50	>50	>50	30.14 ± 0.76	>50
**14**	30.30 ± 1.12	>50	>50	>50	>50
5-FU	32.19 ± 1.03	7.06 ± 0.91	15.13 ± 0.94	12.19 ± 1.10	20.18 ± 1.16

^a^ IC_50_ values are indicated as mean ± S.D of three independent experiments. ^b^ SK-OV-3: human ovarian carcinoma cell line. ^c^ MCF-7: human breast adenocarcinoma cell line. ^d^ MDA-MB-231: human breast adenocarcinoma cell line. ^e^ HCT-116: human colon carcinoma cell line. ^f^ RKO: human colon carcinoma cell line.
